# Prenatal and Lactation Exposure to Bisphenol A (BPA) at the Global Mean Daily Intake Level Leads to Sex-Dependent Changes in Microglial State, Gene Expression, and Behavioral Patterns in Newborn and Juvenile Wistar Rat Offspring

**DOI:** 10.3390/life16091526

**Published:** 2026-09-14

**Authors:** Andrey D. Gerasimov, Alexandra V. Sentyabreva, Elena A. Gantsova, Ekaterina A. Melnikova, Anna M. Kosyreva

**Affiliations:** 1 Avtsyn Research Institute of Human Morphology, Petrovsky National Research Centre of Surgery, 117418 Moscow, Russia; 2 Research Institute of Molecular and Cellular Medicine, Peoples’ Friendship University of Russia (RUDN University), 117198 Moscow, Russia

**Keywords:** bisphenol A, microglia, neural progenitors, behavioral pattern changes, sex differences

## Abstract

Bisphenol A (BPA) is a widespread plasticizer widely used in the food industry worldwide. However, a consensus on the safe level of its daily intake does not currently exist, while there are numerous data pointing to a link between prenatal exposure to BPA and early neuropsychiatric disorders, such as autism spectrum disorder (ASD) and attention deficit hyperactivity disorder (ADHD). The global average human-equivalent dose of BPA is estimated at 30 ng/kg body weight/day. Thus, the research herein was conducted on newborn and juvenile rat offspring of both sexes delivered from rats treated with 30 ng/kg body weight/day orally from day 6 to 21 of pregnancy and for a further 21 days during lactation. Our data indicate that prenatal exposure to 30 ng/kg/day from gestation day 6 to delivery induced sexually dimorphic, region-specific alterations in the neonatal (PD1) rat brains, impacting inflammatory reactions, estrogen receptor signaling, and neural progenitor dynamics. Prenatally BPA-treated males exhibited a pro-inflammatory state characterized by an increased total number of ramified Iba1+ microglia cells in the hippocampus alongside upregulation of *Aif1*, *Nfkb*, *Tnf*, *Il1b*, and *Hif1a*. Conversely, prenatally BPA-treated females showed only an increased total number of hippocampal Iba1+ cells and displayed inflammatory resilience, lacking upregulation of these markers. Both sexes exhibited a pro-inflammatory state in the prefrontal cortex via upregulation of *Nfkb*, *Tnf*, *Il1b*, and *Hif1a*. While both sexes showed decreased *Tgfb* in the prefrontal cortex, they deployed distinct compensatory responses: males upregulated the neuroprotector *Igf1* only in the hippocampus, whereas in females, it was upregulated in both the hippocampus and the prefrontal cortex. Estrogen receptor expression was also highly dimorphic. In the hippocampus, males displayed an imbalance via *Esr1* upregulation and *Esr2* downregulation, while females showed *Esr2* downregulation alone. In the prefrontal cortex, these transcriptional profiles were the opposite. Finally, neural progenitor markers were altered. In the prefrontal cortex, BPA drove the upregulation of *Sox2* and *Pax6* in males but *Sox9* in females; in the hippocampus, only *Pax6* was upregulated, and only in males. The PAX6 protein level in the hippocampus was significantly increased in males but decreased in females. In juvenile offspring (PD21), behavioral patterns characterized by hyper-exploration, increased environmental investigation, and heightened emotional reactivity during social exposure were more pronounced in female offspring. These baseline neonatal disparities followed by behavioral changes underscore the necessity of sex-specific approaches when evaluating early-life neurodevelopment and subsequent adult behavioral and biological vulnerabilities.

## 1. Introduction

Bisphenol A (BPA) is one of the most widely used plasticizers and is a component of a large number of plastic products in the food, medical, and other industries [1]. Its industrial-scale application began approximately 50 years ago, and in 2022, global BPA production exceeded 10 million metric tons [1]. In humans, BPA is detected in urine, blood serum, and breast milk, and it is also deposited in most body tissues, including adipose tissue, the liver, and the brain [2]. The average global daily intake of BPA in humans is estimated at 30 ng/kg body weight [3]. In 2021, the European Food Safety Authority (EFSA) set the tolerable daily intake (TDI) of BPA at 0.2 ng/kg body weight, down from the previously established 50 µg/kg body weight in 2006 and 4 µg/kg body weight in 2015 [4]. However, the US Food and Drug Administration (FDA) still regulates a maximum permissible daily intake of 50 µg/kg body weight [5]. China’s current standards for food contact plastics (National Health Commission of the People’s Republic of China; State Administration for Market Regulation. GB 4806.7-2023: National Food Safety Standard—Food Contact Plastic Materials and Articles; NHC/SAMR: Beijing, China, 2023.) and coatings limit BPA migration (Specific Migration Limit (SML)) to 0.05 mg/kg [6]. There is currently no consensus on a safe daily intake of BPA among these regulatory bodies, which points to an insufficient number of studies on the effects of BPA on various body systems (including during different periods of development) [7,8].

BPA is a synthetic compound structurally similar to the sex steroid hormone estrogen, which allows it to be classified as an endocrine disruptor [9]. Entering the body predominantly from plastics and resins contained in food packaging, BPA interacts with classical [9,10] and non-classical [11] steroid and thyroid receptors, mediating a wide spectrum of effects: the stimulation of adipogenesis, disruption of glucose utilization processes, and the development of insulin resistance, which can potentially lead to metabolic syndrome/obesity and may increase the risk of and lower the age of clinical onset of type 2 diabetes mellitus [12,13,14,15,16]. In addition, BPA induces the formation of reactive oxygen species [17], leads to DNA damage, and prompts epigenetic regulation of the expression of a number of genes, including during prenatal development [18,19], which may initiate carcinogenesis in adults and teratogenesis in embryos [20]. BPA belongs to the category C compounds of high fetal risk [21] and may increase the probability of developing various pathological conditions during different periods of postnatal ontogenesis, including the clinical manifestation of neuroendocrine disorders, as well as attention deficit hyperactivity disorder (ADHD), autism spectrum disorders (ASD), and other neuropsychiatric conditions (thus potentially increasing the risk of neurodegenerative diseases in late ontogenesis) [22,23,24,25]. According to statistics from the US Center for Disease Control and Prevention, the prevalence of ASD has increased significantly over the past twenty years: in 2004, ASD was diagnosed in 1 in 166 children, whereas in 2023, this figure was 1 in 36. Moreover, the prevalence of ASD in boys is 3.8-fold higher than in girls [26]. According to WHO and UNICEF data, 15% of children and adolescents under the age of 18 have diagnosed emotional disorders [27].

A key role in the formation of histo-architectonics and neuroplasticity of the brain during the prenatal period—both under physiological conditions and in the development of pathological processes in postnatal ontogenesis—is played by microglia, the resident immune cells of the CNS [28]. In addition to their surveillance and phagocytic functions [29], microglia also perform non-immunological functions; in particular, they regulate neurogenesis, myelination, and synaptic remodeling during prenatal ontogenesis [30]. A number of studies have also demonstrated that male and female animals show differences in the morphology of microglial cells, their colonization of various brain regions, the expression of a number of genes, and activation under the influence of pro-inflammatory factors [28,30,31,32]. Consequently, changes in the functional activity of microglia during prenatal ontogenesis may lead to disruption of the normal functioning and adaptive processes in neurons and, thereby, contribute to the development of affective and behavioral disorders in early childhood and adolescence, significantly increasing the risk and lowering the age of clinical manifestation of cognitive dysfunction and neurodegenerative processes in late ontogenesis.

Changes in the morphofunctional features of microglia occur upon the activation of various receptors. Microglial cells express a wide spectrum of steroid receptors, including ERα and ERβ estrogen receptors, as well as androgen, mineralocorticoid, and glucocorticoid receptors [33]. Consequently, BPA may trigger signaling cascades via activation of steroid receptors, leading to the further realization of its effects. Experimental studies in rats have shown that offspring (predominantly males) born to females that consumed BPA during pregnancy at dosage from 0.5 to 50 mg/kg body weight/day exhibited impairments in learning and memory processes—in particular, spatial memory—as well as in sociosexual behavior, alongside a reduction in exploratory activity [34,35]. It has also been shown that culturing the BV2 microglial cell line in the presence of BPA resulted in enhanced mRNA expression and protein levels of the pro-inflammatory cytokines TNF and IL-6 [36]; at the same time, addition of BPA to the HT-22 hippocampal cell line and primary neuronal cultures revealed increased levels of the transcription factor NF-κB protein, which activates the inflammatory response, and increased production of reactive oxygen species [37]. It has also been demonstrated that direct exposure of DNA to BPA leads to epigenetic changes in the expression of a number of genes in various cell populations, including those associated with nervous system development (e.g., *Fkbp5*, *Grin2b*, *Bdnf*, *Era*, *Kcc2*, and others) [38]. In recent studies, gene methylation has been identified as an important marker of cellular aging, including premature aging [39,40,41]. Therefore, BPA exposure during the late prenatal period and early postnatal ontogenesis might possibly lead to morphofunctional changes in microglial cells, including persistently sustained pro-inflammatory activation, potentially increasing the risk of neurological and mental disorders in children during early postnatal ontogenesis.

Despite the enormous body of literature on the effects of BPA on estrogen-dependent organs, studies dedicated to examining the effects of BPA on the immune system and CNS are few in number and have been conducted predominantly on male offspring [42,43,44]. In most studies, results have been obtained across a wide range of BPA doses, often markedly exceeding the amount of BPA—from 0.05 to 50 mg/kg/body weight/day [34,35,38]—that enters the human body and is permitted by international regulatory agencies [4,6]. Therefore, studies of the effects of low-dose BPA consumption during pregnancy on the different brain regions in a sex-dependent manner are highly relevant. The aim of this exploratory study was to investigate potential neurobiological and behavioral alterations associated with prenatal and postnatal BPA exposure in offspring of both sexes, with particular emphasis on microglial responses and pro-inflammatory signaling in neonatal rats and social behavior during early postnatal development.

## 2. Materials and Methods

### 2.1. Animals

This study was performed on Wistar rat offspring of both sexes born to females that received BPA during pregnancy. Female (*n* = 12) and male (*n* = 18) Wistar rats (3 months old) were purchased from the Stolbovaya branch of the Federal State Budgetary Scientific Institution National Center for Biomedical Technologies of the Federal Medical-Biological Agency of Russia (FSBSI NCBT FMBA Russia) and acclimatized for 2 weeks prior to the start of the experiment. Animals were housed in plastic cages (60 cm × 38 cm × 18.5 cm) in social groups of five individuals of the same sex. The vivarium room temperature was maintained within 18–22 °C, and air humidity was 50–65%. The light cycle was 12:12 h (lights on at 07:00). Laboratory animal food (PK-120-1, Laboratorysnab LLC (Moscow, Russia), certificate of conformity No. POCCRU.nO81.B00113, GOST R 50258-92) and drinking water were available ad libitum. Experimental protocols were approved by the Bioethical Committee of the Federal State Budgetary Scientific Institution “B.V. Petrovsky National Medical Research Centre of Surgery” (Protocol No. 10 dated 21 November 2025). All procedures involving experimental animals were carried out in accordance with the principles of the European Convention for the Protection of Vertebrate Animals Used for Experimental and Other Scientific Purposes (ETS 123, Strasbourg, 1986), and the Directive of the European Parliament and of the Council of the European Union (2010/63/EU, Strasbourg, 2010).

### 2.2. Mating and BPA Administration

Female rats were co-housed with males at a 2:1 ratio for 16 h (4 p.m.–8 a.m.). The day for mating was selected according to the estrous cycle, when females were in proestrus (according to the colpocytology). Pregnancy was confirmed on day 6 after mating by ultrasound examination using a Mindray DC-40 device (Shenzhen, China). Pregnant females were randomly divided into 2 groups: control (*n* = 5) and BPA 30 ng/kg body weight/day (*n* = 7).

Bisphenol A (D5921, Tokyo Chemical Industry Co., Ltd., Tokyo, Japan) was dissolved in 96° ethanol to a concentration of 10 µg/mL (stock solution). BPA stock solution (22 µL) was added to 5 mL of 0.5% glucose solution to achieve a concentration of 30 ng/mL in 200 µL of working solution, so the final concentration of ethanol was at trace level (approximately 0.66 µg per 200 µL of daily administration). Fresh solution was prepared every 5 days. The dose of 30 ng/kg corresponds to the average daily BPA intake in humans [3]. Control group females received 200 µL of 0.5% glucose solution daily via gavage from gestational day 6 to day 21 (until delivery). Experimental group females received BPA orally at a dose of 30 ng/kg body weight/day in 200 µL of 0.5% glucose solution via gavage from gestational day 6 to day 21 (until delivery). One pup of each sex from one delivered female included in control and experimental groups was selected randomly. The following groups were included in the subsequent study: MC—male control (*n* = 5); FC—female control (*n* = 5); MBPA—males born to females that received BPA at 30 ng/kg body weight/day from gestational days 6 to 21 (*n* = 7); and FBPA—females born to females that received BPA at 30 ng/kg body weight/day from gestational days 6 to 21 (*n* = 7).

After delivery, BPA administration to delivered rats was continued during the lactation from PD0 to PD21; then, juvenile offspring were included in the behavioral study.

Different researchers were involved in the experiment. E.A.M. performed BPA administration; K.A.M. randomly divided the animals into groups and coded the samples; and E.A.G., A.V.S., and A.D.G. performed histological, ELISA, and molecular studies, while the group assignment of the samples was not known.

### 2.3. Biological Sample Collection

On the day following birth (postnatal day 1, PD1), offspring of both sexes—born to control females and to females that received BPA during pregnancy—were anesthetized and peripheral blood samples were collected, after which they were euthanized by Zoletil overdose (15 mg/kg) (Vibrac Santé Animale, Carros, France). Whole brains were extracted, and all subsequent manipulations were performed on ice. The left hemisphere was fixed in 10% buffered formalin solution (BioVitrum, Saint Petersburg, Russia) for 48 h, after which it was placed in 70° ethyl alcohol. The left cerebral hemisphere was then processed through ascending concentrations of alcohols in an automated tissue-processing apparatus Tissue-Tek VIP5Jr (Sakura, Tokyo, Japan) and embedded in histomix. Histological sections of 5–7 µm thickness were cut using a Microm HM340E microtome (Thermo Scientific, Waltham, MA, USA). From the right hemisphere, a fragment of the hippocampus and the prefrontal cortex was dissected and fixed in IntactRNA (Eurogen, Moscow, Russia) for subsequent RT-qPCR analysis. Blood samples were also obtained from the tail vein of delivered rats euthanized by Zoletil (4 mg/kg) (Vibrac Santé Animale, Carros, France).

### 2.4. Enzyme-Linked Immunosorbent Assay (ELISA)

To determine BPA levels in the blood serum of PD1 male and female rat offspring from control groups and from females that received BPA at 30 ng/kg body weight/day from gestational days 6 to 21, an enzyme-linked immunosorbent assay was performed using a commercial kit for detection of free BPA (E01B1032, BlueGene, Shanghai, China), with a minimum sensitivity of 1 ng/mL. To assess the pro-inflammatory state of delivered rats due to BPA consumption, calprotectin level was determined using a commercial kit (ER1636, FineTest, Wuhan, China). Optical density was assessed using an AMR-100 microplate spectrophotometer (Allsheng, Hangzhou, China).

### 2.5. Immunohistochemical and Morphometric Analysis

For immunohistochemistry (IHC-P), frontal brain histological sections at the level of the hippocampus and lateral ventricles were prepared (2 mm dorsal to bregma). Histological preparations were deparaffinized according to a standard protocol, and antigen retrieval was performed in citrate buffer pH 6.0 with 0.5% Tween-20 at 100 °C. After washing in phosphate-buffered saline (PBS, pH 7.2–7.4), endogenous peroxidase was blocked with 3% H_2_O_2_ solution, followed by blocking with protein buffer (PBS with 0.1% bovine serum albumin) at room temperature for 30 min to minimize non-specific antibody binding. Primary anti-Iba1 antibodies (1:100, PAC288Ra01, CloudClone, Wuhan, China) were used. Sections were incubated with primary antibodies at +4 °C overnight. The following day, after washing in phosphate-buffered saline, secondary Carpine-anti-Rabbit HRP antibodies (1:500, SAA544Rb19, CloudClone, Katy, TX, USA) were applied and incubated at room temperature for 2 h. HRP labels of secondary antibodies were visualized using DAB and counterstained with hematoxylin. Images were obtained using a Zeiss Axioplan 2 microscope and an AxioCam HRc camera (Germany) at ×640 magnification. The total number of Iba1+ cells (along with the number of ramified and amoeboid Iba1+ cells per 1 mm^2^) was manually counted on 3 sections per animal (and the mean numbers from 3 sections were calculated) using the stained sections digitized with a KF-PRO-005 histoscanner (KFBIO, Yuyao, China). Section areas were determined using QuPath v.0.6.0 software [45] in stereotaxic coordinates according to a P1 Atlas of the rat brain [46].

### 2.6. RT-qPCR Analysis

mRNA expression was analyzed by RT-qPCR in the prefrontal cortex and hippocampus fragments (*n* = 5 for control groups and *n* = 7 for prenatally BPA-treated groups). For mRNA isolation, the ExtractRNA reagent (Eurogen, Moscow, Russia) was used; reverse transcription was performed using the MMLV RT Kit (Eurogen, Russia). Amplification using qPCRmix-HS SYBR (Eurogen, Russia), containing the fluorescent intercalating dye SYBR Green I, was carried out on a real-time DTprime amplifier (DNA-Technology, Moscow, Russia). A single, distinct melting peak was confirmed for each primer pair, confirming amplification specificity and the absence of primer-dimers or non-specific amplification products. Primer amplification efficiency was determined by standard curve analysis and was 98% (E = 1.98; slope = −3.371). The following mRNA expression levels were determined: the pan-microglial marker *Aif1*; estrogen receptors *Esr1* and *Esr2*; markers of neural progenitor cells *Sox2*, *Sox9*, and *Pax6*; markers of synaptic contact formation and myelination *App* and *Bace1*; pro-inflammatory markers *Nfkb*, *Tnf*, and *Il1b*; the anti-inflammatory cytokine *Tgfb*; the hypoxia-inducible factor *Hif1a*; and the neuroprotective marker *Igf1*. Measured mRNA expression levels were normalized to the expression level of the housekeeping gene *Gapdh* [47]. In all samples in the control and experimental groups, *Gapdh* expression was recorded at 21–22 cycles; thus, it was proved stable. For gene expression analysis, the threshold cycle (Ct) method was employed, and relative gene expression was calculated using the method proposed by Pfaffl M.W. [48]. Each biological sample was analyzed in technical triplicate, and the mean Ct value were used for calculation. The relative mRNA concentrations of the specified genes were determined via direct data comparison using the formula [A]0/[B]0 = EΔCt, where [A]0 represents the initial mRNA concentration of the target gene in the PCR mixture; [B]0 represents the initial mRNA concentration of GAPDH in the PCR mixture; E denotes the reaction efficiency (assumed to be 1.98); and ΔCt represents the difference between the threshold cycles of GAPDH and the target gene, calculated using Microsoft Office Excel 2016. For better calculation and visualization, the final relative expression result was multiplied by 10^5^ and visualized on the graphs as ×10^−5^. Primers were designed using Primer-BLAST software (version 2.5.0) (USA) and were species-specific for *Rattus norvegicus* (Supplement S1).

### 2.7. Western Blotting (WB) Analysis

Fragments of the hippocampus (3 independent samples per group) were lysed in protein solubilization buffer (PSB) from MicroRotofor™ Cell Lysis Kit (Mammal) (#1632141, Bio-Rad Laboratories, Hercules, CA, USA) and centrifuged at 11,500× *g* and +4 °C for 30 min. The supernatant was mixed in a proportion of 1:1 with 2× Laemmli Sample Buffer c β-mercaptoethanol and heated for 5 min at +65 °C. Protein separation was carried out in 4–15% sodium dodecyl sulfate–polyacrylamide gel electrophoresis (SDS-PAGE). After electrophoresis, the proteins from the gel were transferred to a polyvinylidene difluoride (PVDF) membrane in the Trans-Blot Turbo Transfer System (Biorad, USA) at 25 V, 1 A for 45 min. After transfer, the membranes were washed with deionized water, and non-specific binding sites were blocked using a 5% solution of Blotting-Grade Blocker (#1706404, Biorad, USA) at room temperature for 1 h. Then, the membranes were incubated for 24 h at +4 °C in a solution of primary antibodies: rabbit anti-PAX6 1:1000 (DF7557, Affinity Biosciense, Liyang, Jiangsu, China) and rabbit anti-GAPDH 1:5000 (Cat. No. 5G4, clone 4G5, HyTest, Moscow, Russia). After incubation with primary antibodies, membranes were washed in TBST and incubated at room temperature for 1 h in a solution of secondary antibodies: goat–anti-rabbit IgG-HRP 1:5000 (ab6721, Cambridge, UK, Abcam). After incubation with secondary antibodies, membranes were washed in TBST and developed using the Clarity Western ECL substrate (#1705060, Biorad, USA). The chemiluminescence signal was detected on a ChemiDoc Imaging System (Bio-Rad Laboratories, Hercules, CA, USA) and Image Lab Touch Software (version 3.0.1) (Bio-Rad Laboratories, Hercules, CA, USA). The GAPDH protein was used as a reference protein for application; the signals of target proteins were normalized to the signal level of this protein, and target protein levels were evaluated by adjusted volume relative to one of the reference proteins.

### 2.8. Behavioral Test for Preference and Novelty in Juvenile Offspring

A behavioral study (three-chamber test) used for ASD models [49] was conducted to evaluate social preference and novelty in juvenile (21 days old) male and female rats of the control groups as well as those born to females treated with BPA at a dose of 30 ng/kg body weight/day from days 6 to 21 of gestation and for 21 day after birth via lactation. For the behavioral study, 2 males and 2 females were taken from the same litters as previous newborn offspring. The data from same-sex offspring per litter were averaged. Thus, the final statistics were calculated from *n* = 5 for control groups and *n* = 7 for treated groups. Animals were placed individually in the center of a three-chamber arena consisting of three compartments connected by passages. The right compartment contained a small cage with an «unfamiliar» rat (from a different cage) of the same sex as the test animal; the left compartment contained a cage with a sibling «familiar» rat (from the same cage) of the same sex as the test animal. The behavior of the test rat was recorded for 10 min, including the number of entries into different compartments, the time spent in different compartments, the number of rearings, and grooming episodes.

### 2.9. Statistical Analysis

Statistical analysis was performed using GraphPad Prism 8.1 (GraphPad Software, USA) and R software (version 4.6.1; R Foundation for Statistical Computing, Vienna, Austria). To avoid pseudoreplication, one male and one female offspring were randomly selected from each litter at PD1. For behavioral study at PD21 2 males and 2 females were taken from the same litters as newborn offspring before. The data from same-sex offspring per litter were averaged. So, the final statistics was calculated from *n* = 5 for control groups and *n* = 7 for treated groups. Males and females were analyzed separately, so, each litter was represented by only one independent observation of each sex. The data were tested for normal distribution using the Shapiro–Wilk test. Since the data were not normally distributed, significance of differences between groups was assessed using the non-parametric two-factor Scheirer–Ray–Hare test and post-hoc Dunn’s test; multiple comparisons were verified using the Benjamini–Hochberg procedure applied based on the methods used (ELISA, IHC, qPCR-RT, WB, behavioral study). Hypothesis families are as arranged in the manuscript figures. Behavioral hypotheses were divided on social preferences and exploratory behavior. The Mann-Whitney U-test was used for pair comparison between control and BPA-treated delivered rats. The chi-square test with Yates’s correction for contingency verified using the Benjamini–Hochberg (BH) procedure was used to test the relationship between two categorical features (grooming episodes in the behavior study). Differences between groups were considered statistically significant at *p* < 0.05. *p*-values represented in figures were adjusted using the BH procedure.

## 3. Results

### 3.1. Serum BPA Levels Did Not Differ Between the Offspring of Control and BPA-Treated Rats, While Calprotectin Differed Between Control and BPA-Treated Delivered Rats

**Figure 1:**
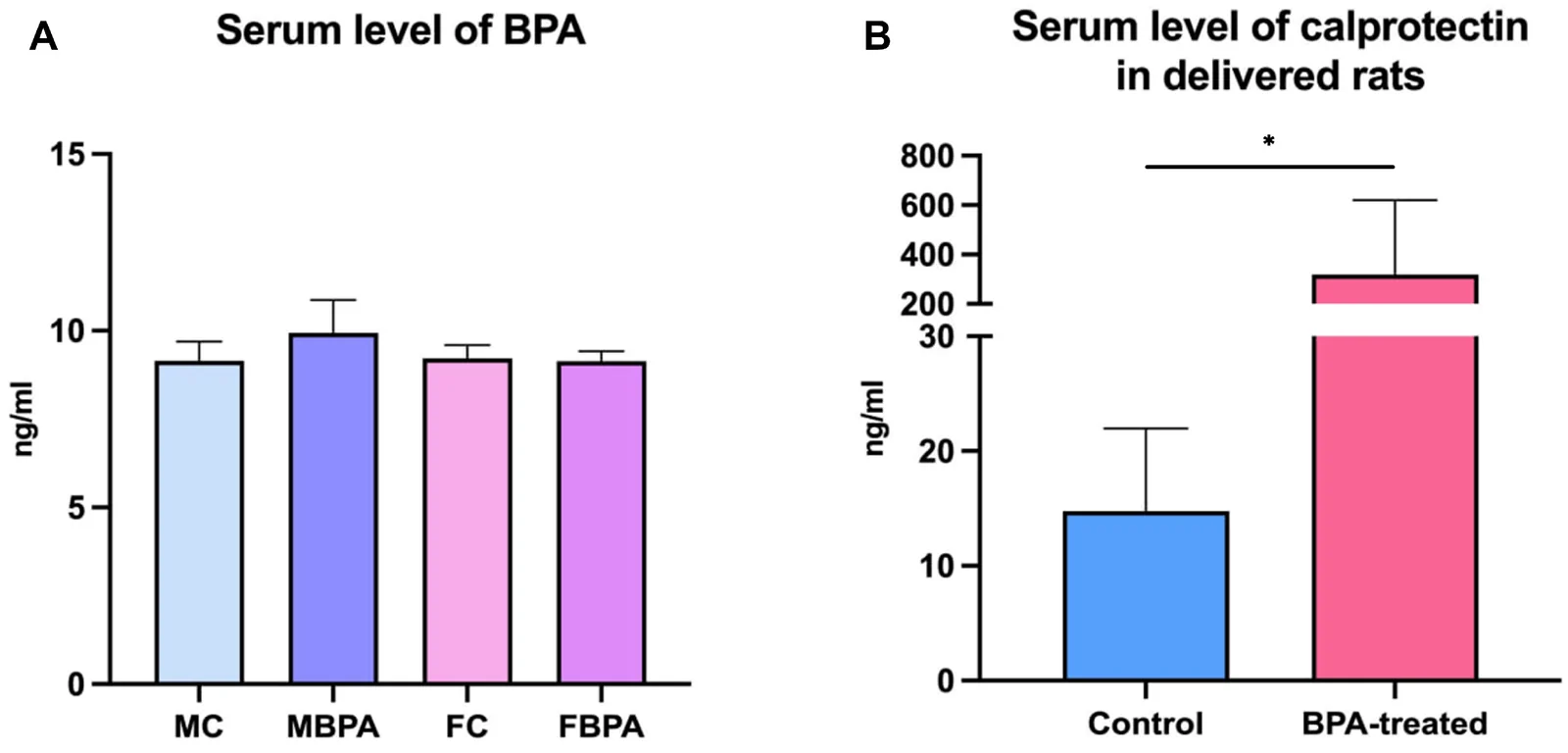
Free BPA levels in blood serum of newborn male and female rats from control and experimental groups (**A**) and calprotectin levels in blood serum of control and BPA-treated delivered rats (**B**). MC—males born to control rats; FC—females born to control rats; MBPA—males born to females that received BPA at 30 ng/kg body weight/day from gestational days 6 to 21; FBPA—females born to females that received BPA at 30 ng/kg body weight/day from gestational days 6 to 21. *—*p* < 0.05. Data are presented as Me (LQ 25%; UQ 75%). The Mann-Whitney U-test was used for pair comparison.

When assessing BPA levels in the blood serum of offspring of both sexes born from control rats and from rats that received BPA at 30 ng/kg body weight/day from gestational days 6 to 21, no statistically significant differences were found between any of the groups studied (Figure 1A). The detectable signal recorded during optical density measurement by spectrophotometry is likely attributable to cross-reactivity with estrogen, whose structure is similar to that of BPA. Given that BPA was administered to pregnant female rats at an extremely low dose, it was likely metabolized and bound to proteins such as albumin and therefore passed through the placental barrier in its free form only to a negligible extent. Previously, it was shown that after oral consumption of BPA by pregnant rats at a dose of 5 µg/kg body weight/day, BPA was detected in the offspring’s serum at levels of 4–5 ng/mL [50]. In our experiment, females consumed 30 ng/kg body weight/day. Consequently, BPA is most likely present in the offspring’s serum at picogram levels, which falls below the detection limit of most ELISA kits and high-performance liquid chromatography methods. However, to investigate the effect of BPA, serum levels of calprotectin were assessed in delivered rats; its concentration was markedly higher in BPA-treated females (Figure 1B). Serum calprotectin is concidered as a biomarker of systemic inflammation [51]. Therefore, maternal inflammatory activation was suggested due to increased calprotectin levels.

### 3.2. Prenatal BPA Exposure Altered the Number and Morphology of Iba1+ Microglial Cells and the Expression Level of Aif1 in a Sex-Dependent Manner

**Figure 2:**
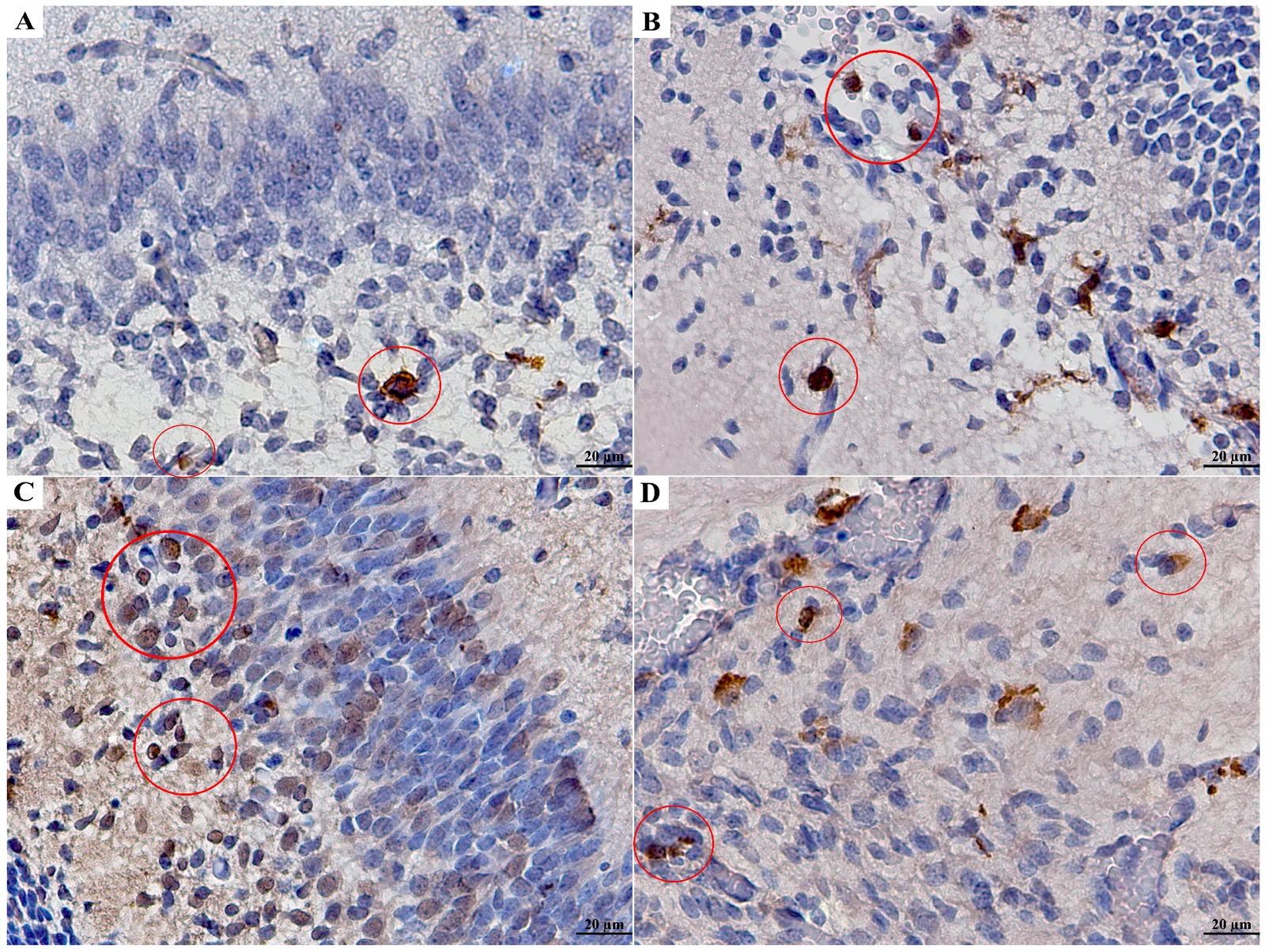
Amoeboid Iba1+ microglial cells in the hippocampus of newborn male and female rats from control and experimental groups: (**A**) males born to control rats; (**B**) females born to control rats; (**C**) males born to females that received BPA at 30 ng/kg body weight/day from gestational days 6 to 21; (**D**) females born to females that received BPA at 30 ng/kg body weight/day from gestational days 6 to 21. Primary anti-Iba1 antibodies + secondary Carpine-anti-Rabbit HRP conjugated antibodies counterstained with hematoxylin are shown at ×640.

**Figure 3:**
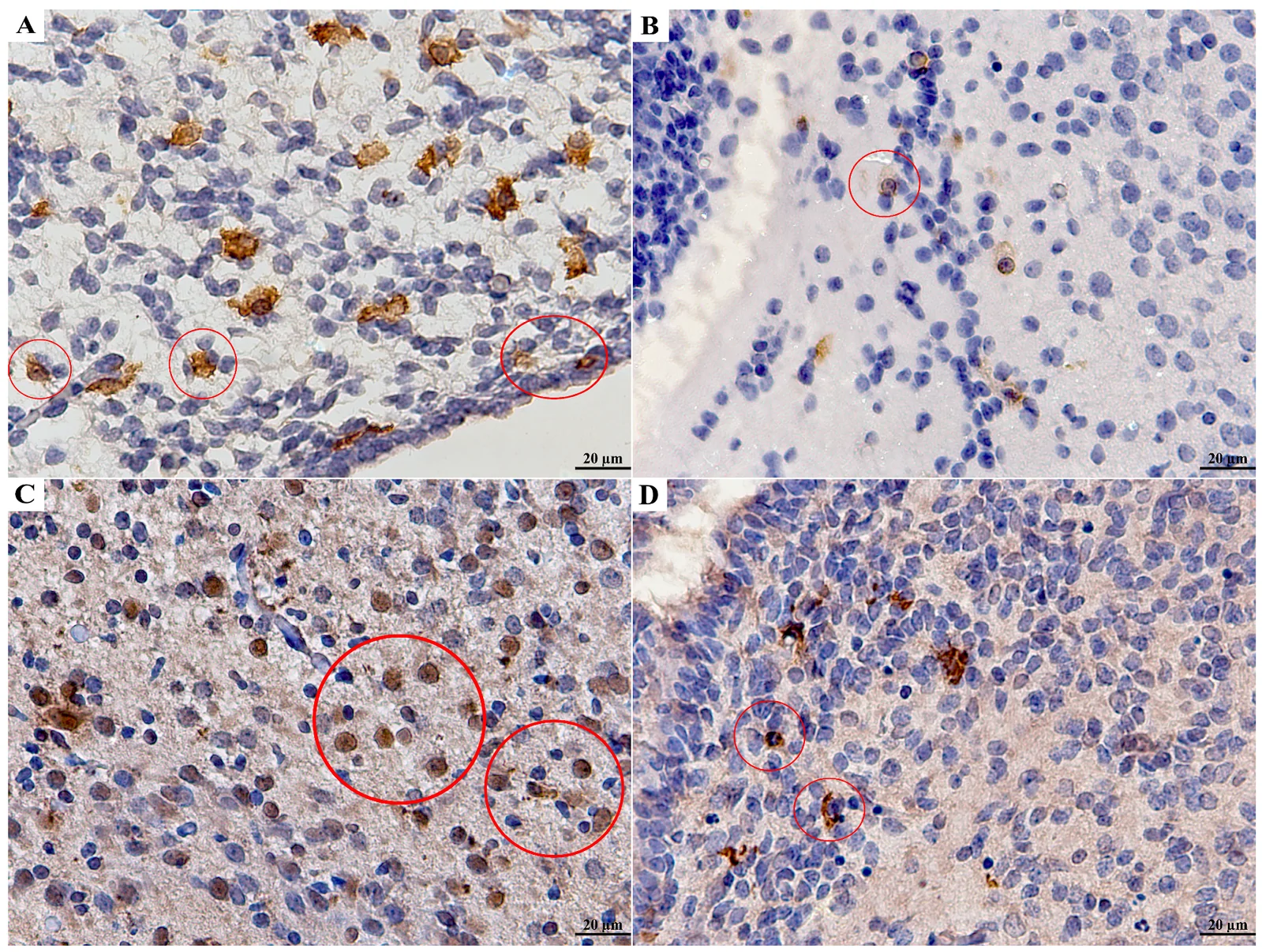
Amoeboid Iba1+ microglial cells in the periventricular zone of newborn male and female rats from control and experimental groups: (**A**) males born to control rats; (**B**) females born to control rats; (**C**) males born to females that received BPA at 30 ng/kg body weight/day from gestational days 6 to 21; (**D**) females born to females that received BPA at 30 ng/kg body weight/day from gestational days 6 to 21. Primary anti-Iba1 antibodies + secondary Carpine-anti-Rabbit HRP conjugated antibodies are shown counterstained with hematoxylin at ×640.

**Figure 4:**
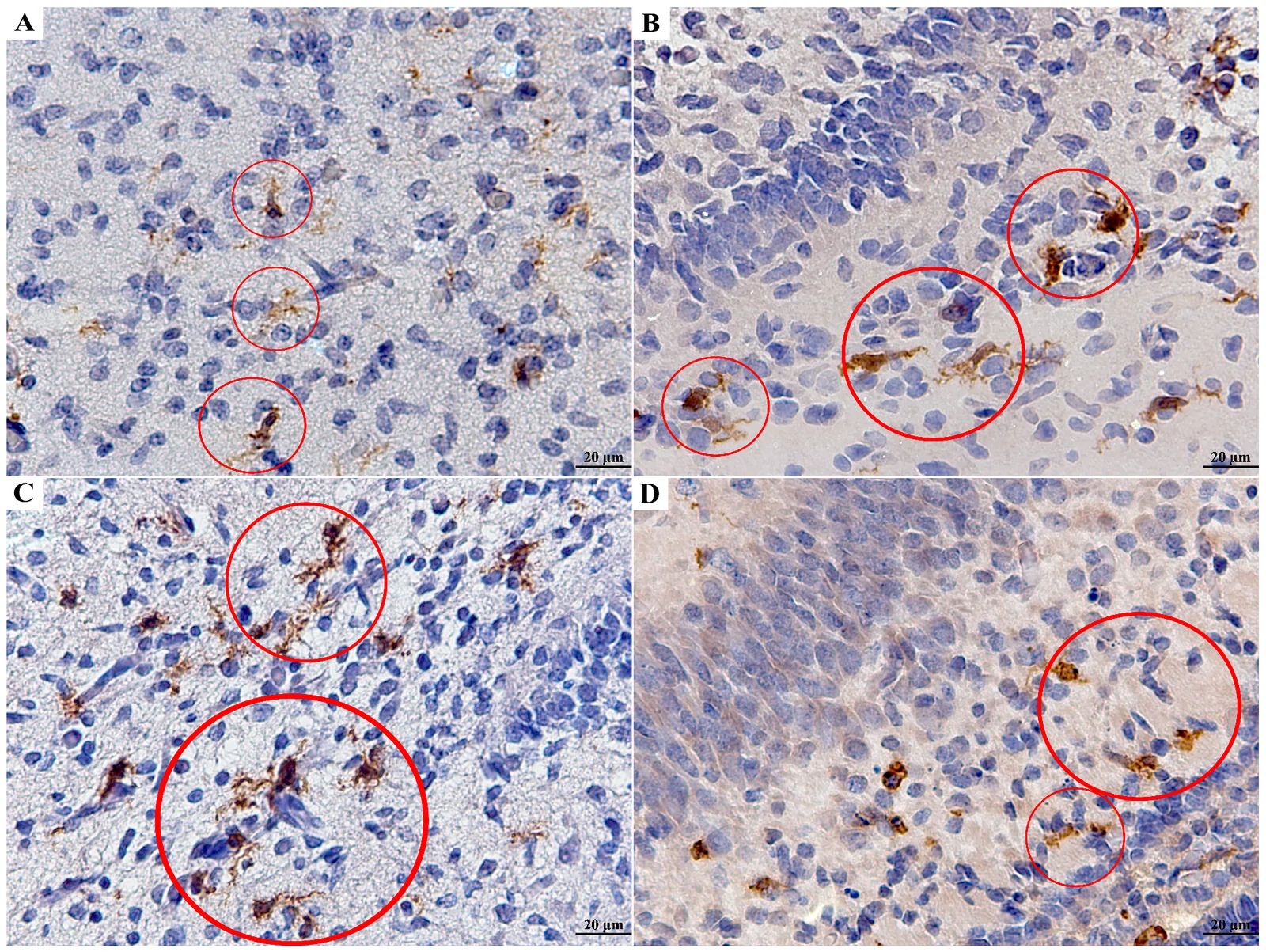
Ramified Iba1+ microglial cells in the hippocampus of newborn male and female rats from control and experimental groups: (**A**) males born to control rats; (**B**) females born to control rats; (**C**) males born to females that received BPA at 30 ng/kg body weight/day from gestational days 6 to 21; (**D**) females born to females that received BPA at 30 ng/kg body weight/day from gestational days 6 to 21. Primary anti-Iba1 antibodies + secondary Carpine-anti-Rabbit HRP conjugated antibodies are shown counterstained with hematoxylin at ×640.

**Figure 5:**
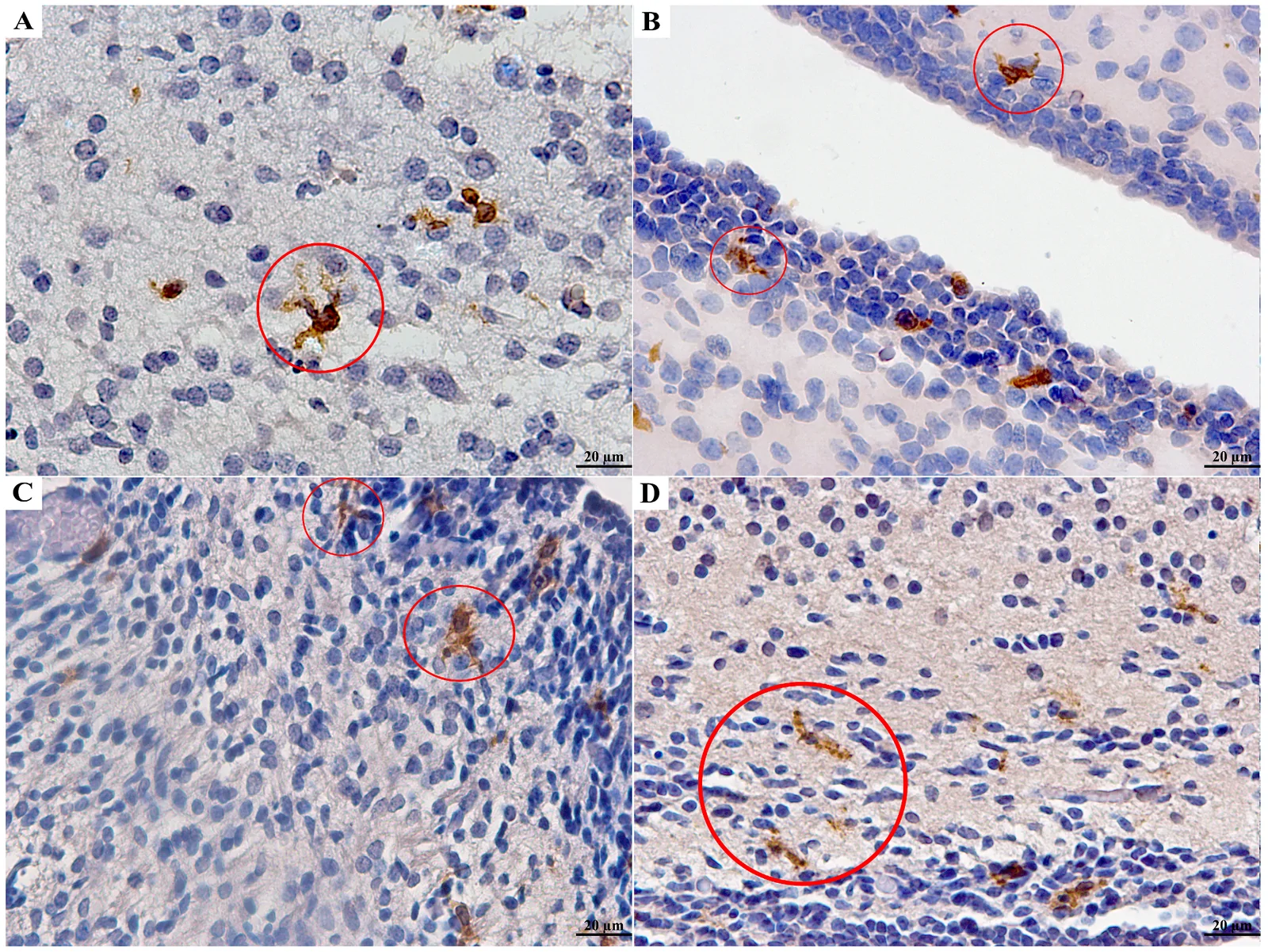
Ramified Iba1+ microglial cells in the periventricular zone of newborn male and female rats from control and experimental groups: (**A**) males born to control rats; (**B**) females born to control rats; (**C**) males born to females that received BPA at 30 ng/kg body weight/day from gestational days 6 to 21; (**D**) females born to females that received BPA at 30 ng/kg body weight/day from gestational days 6 to 21. Primary anti-Iba1 antibodies + secondary Carpine-anti-Rabbit HRP conjugated antibodies are shown counterstained with hematoxylin at ×640.

**Figure 6:**
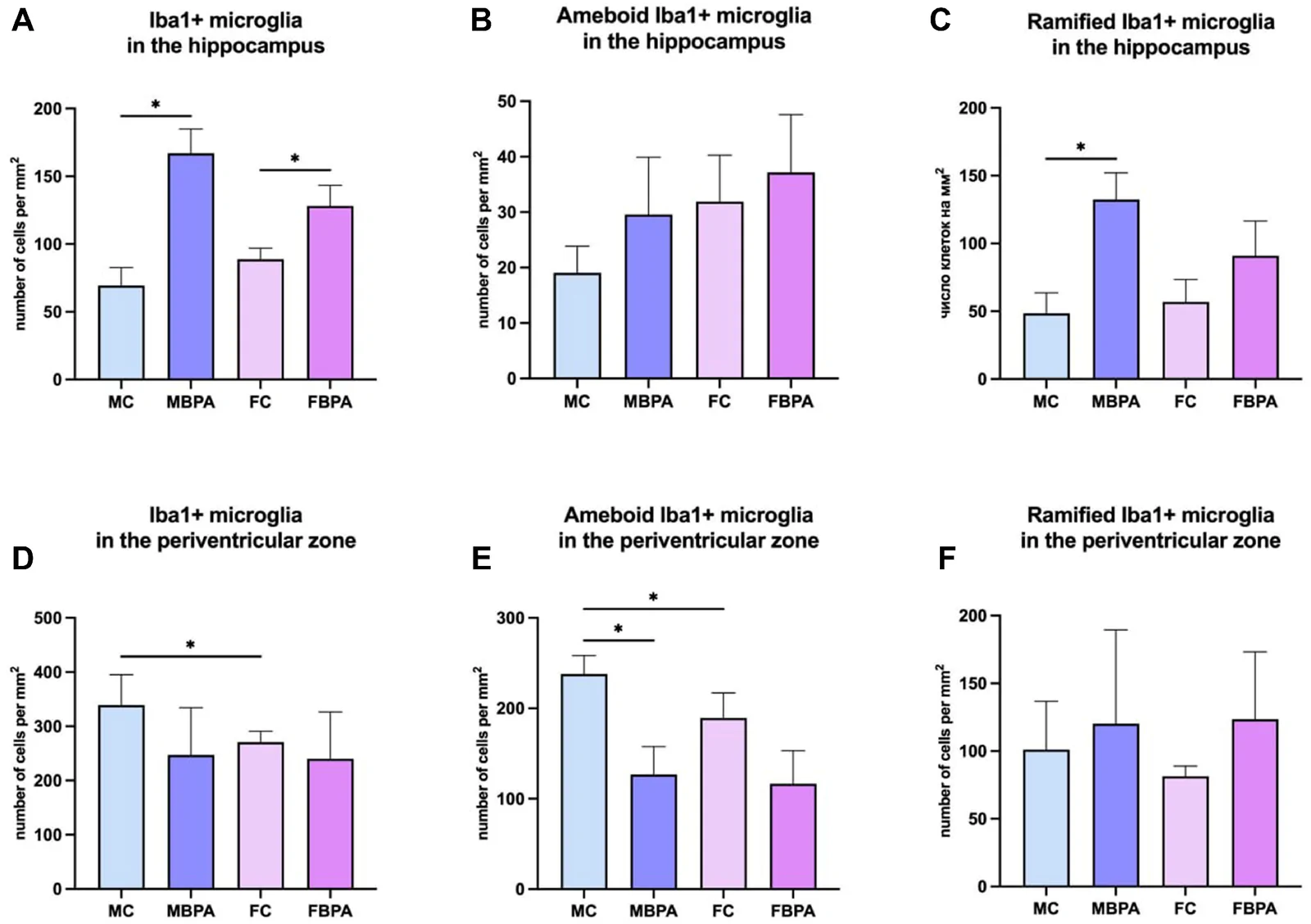
Total number of Iba1+ cells (**A**,**D**), ramified (**B**,**E**), and amoeboid (**C**,**F**) Iba1+ cells in the hippocampus (**A**–**C**) and periventricular zone (**D**–**F**) in control and experimental groups of newborn male and female rats. MC—males born to control rats; FC—females born to control rats; MBPA—males born to females that received BPA at 30 ng/kg body weight/day from gestational days 6 to 21; FBPA—females born to females that received BPA at 30 ng/kg body weight/day from gestational days 6 to 21. *—*p* < 0.05. Data are presented as Me (LQ 25%; UQ 75%).

To assess the effect of BPA on CNS immune cells, we counted the total number of Iba1+ cells in the hippocampus and periventricular zone and, separately, the number of amoeboid microglial cells of rounded shape with shortened processes (Figure 2 and Figure 3) and ramified microglial cells with long branching processes (Figure 4 and Figure 5). No sex differences in the total number of Iba1+ cells in the hippocampus were detected either between control or experimental groups. At the same time, the total number of Iba1+ microglial cells was increased in both experimental groups compared to the corresponding controls in the hippocampus (Figure 2, Figure 4 and Figure 6). The number of amoeboid Iba1+ microglial cells did not differ between any of the groups. However, males that underwent prenatal BPA exposure had significantly more ramified microglial cells compared to controls, whereas no differences were observed between control and experimental females. No sex differences in the number of ramified Iba1+ cells in the hippocampus were detected (Figure 6).

In contrast to the hippocampus, in the periventricular zone, which is considered a progenitor niche, the number of microglial cells in experimental offspring of both sexes showed a tendency toward reduction. The total number of Iba1+ microglial cells in the periventricular zone was higher in control males than in control females, whereas no sex differences were found between the experimental groups. No differences between control and experimental groups were found for either sex. However, sex differences were identified between control offspring groups: the number of amoeboid Iba1+ microglial cells in the periventricular zone was significantly higher in control males. Prenatally BPA-treated males also showed a lesser number of amoeboid Iba1+ cells compared to their experimental counterparts. The number of ramified microglial cells did not differ between any of the groups (Figure 3, Figure 5 and Figure 6).

**Figure 7:**
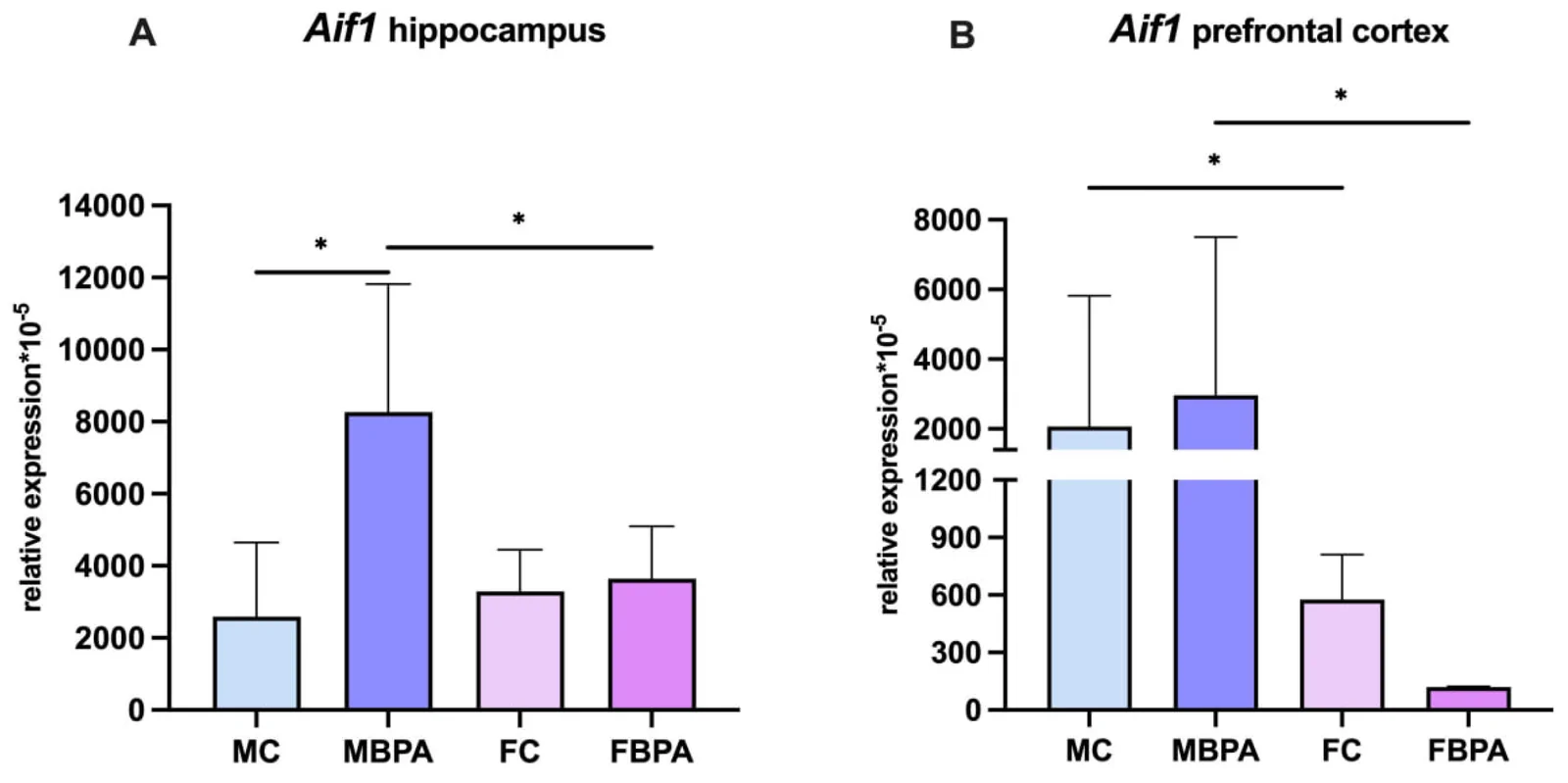
mRNA expression levels of *Aif1* in the hippocampus (**A**) and the prefrontal cortex (**B**) of control and experimental newborn male and female rats. MC—males born to control rats; FC—females born to control rats; MBPA—males born to females that received BPA at 30 ng/kg body weight/day from gestational days 6 to 21; FBPA—females born to females that received BPA at 30 ng/kg body weight/day from gestational days 6 to 21. *—*p* < 0.05. Data are presented as Me (LQ 25%; UQ 75%).

*Aif1* is the gene encoding the calcium-binding protein Iba1, which is highly expressed in macrophages and, in particular, microglia [52]. In the hippocampus, its expression was almost 4-fold higher in prenatally BPA-treated male offspring relative to controls, which corresponded to an increase in Iba1+ cells in the hippocampus of experimental males. Moreover, *Aif1* expression was also higher in the experimental male group than females (Figure 7A). Meanwhile, sex differences between control groups were found in *Aif1* expression levels in the prefrontal cortex: expression of *Aif1* in control males was 3.6-fold higher than control females, and in males that underwent prenatal BPA exposure, it was nearly 26-fold higher than in experimental females. No differences in expression of this gene were found between control and experimental offspring of both sexes, despite a tendency toward an increase in experimental male and a decrease in experimental female offspring (Figure 7B).

### 3.3. Prenatal Exposure to BPA Led to Differences in Estrogen Receptor Expression Depending on Brain Region

To assess the prenatal effects of BPA on estrogen receptors in the hippocampus and prefrontal cortex, we analyzed mRNA expression levels of *Esr1* and *Esr2* genes, which encode estrogen receptors α and β, respectively.

**Figure 8:**
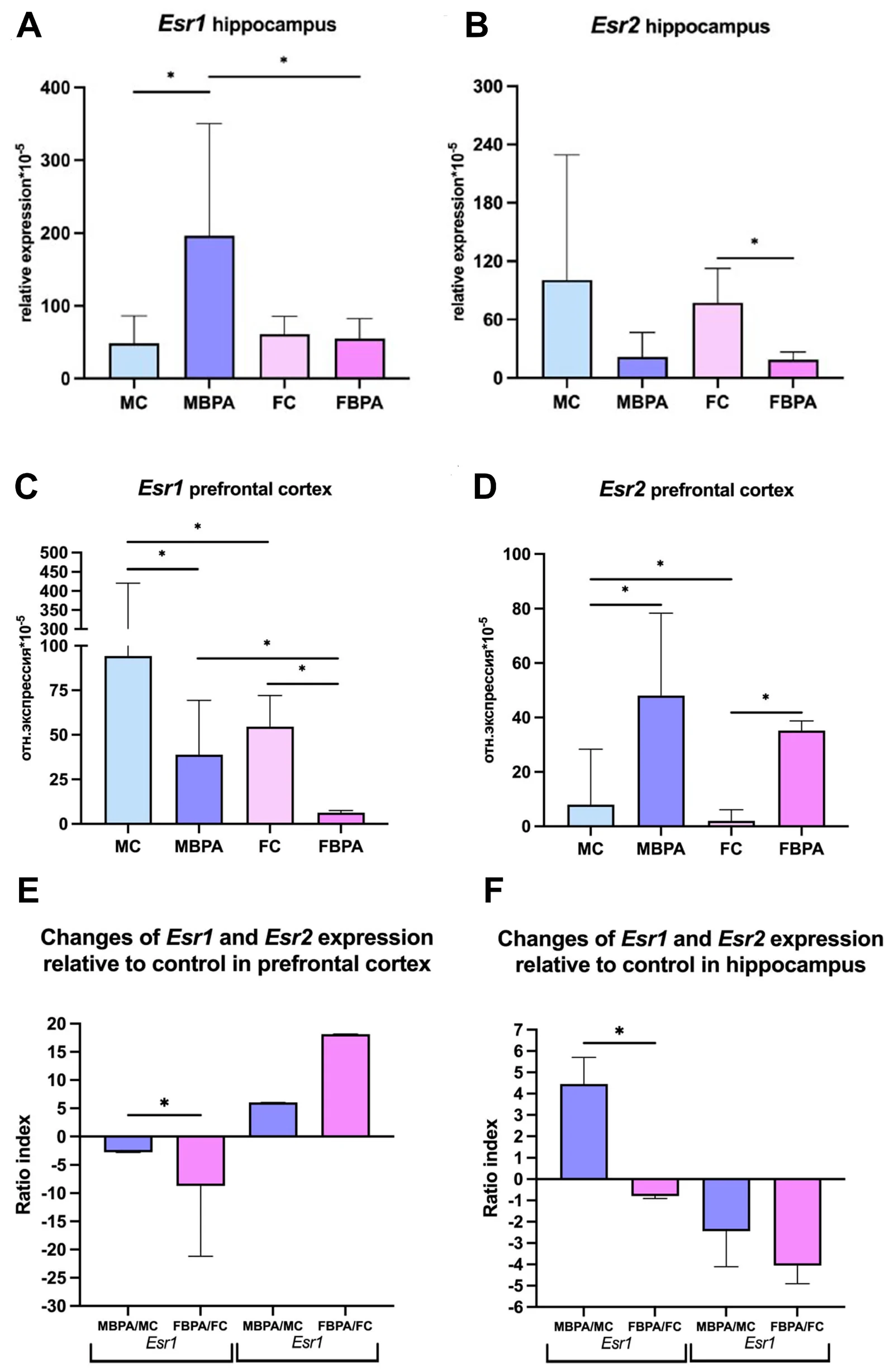
mRNA expression levels of estrogen receptors *Esr1* (**A**,**C**) and *Esr2* (**B**,**D**) in the hippocampus (**A**,**B**) and prefrontal cortex (**C**,**D**) of control and experimental newborn male and female rats and their ratio index in the hippocampus (**E**) and prefrontal cortex (**F**). MC—males born to control rats; FC—females born to control rats; MBPA—males born to females that received BPA at 30 ng/kg body weight/day from gestational days 6 to 21; FBPA—females born to females that received BPA at 30 ng/kg body weight/day from gestational days 6 to 21. *—*p* < 0.05. Data are presented as Me (LQ 25%; UQ 75%).

According to the literature, most cells in the CNS, including microglia, express a wide spectrum of steroid receptors, including estrogen receptors ERα and ERβ [33]. In binding to them, BPA potentially might trigger signaling cascades, leading to the further realization of its pro-estrogenic effects. In the hippocampus, there were no differences between control male and female offspring, while in prenatally BPA-treated males, its expression was 2-fold higher than in the corresponding female group (Figure 8A). Moreover, *Esr1* expression was upregulated in experimental males relative to controls. At the same time, *Esr2* was downregulated only in prenatally BPA-treated females and relative to control female offspring, while no other differences were discovered (Figure 8B). Interestingly, the opposite dynamics were found in the prefrontal cortex. Thus, in offspring of both control and BPA-treated pregnant rats, sex differences were identified: *Esr1* expression levels were higher in males. At the same time, in experimental offspring of both sexes, its expression was significantly lower than in controls (Figure 8C). Similar sex differences were identified in the expression level of the estrogen receptor *Esr2*, which encodes the ERβ receptor protein, in the control groups. Its expression, like *Esr1*, was higher in males. No sex differences in *Esr2* expression levels were observed between experimental groups. In both males and females, *Esr2* expression was significantly upregulated in offspring that underwent prenatal BPA exposure compared to control groups (Figure 8D). The effects of prenatal BPA exposure were predominantly unidirectional, with the exception of upregulated *Esr1* expression in the experimental male group compared to corresponding females in the hippocampus. In females of the experimental group, the decrease in *Esr1* expression was observed in both investigated regions and was more pronounced in the prefrontal cortex relative to prenatally BPA-treated males (Figure 8E,F).

### 3.4. Prenatal Exposure to BPA Resulted in Changes in Pro-Inflammatory Gene Expression and Neuroprotective Markers Unidirectionally in Both Sexes of Offspring

**Figure 9:**
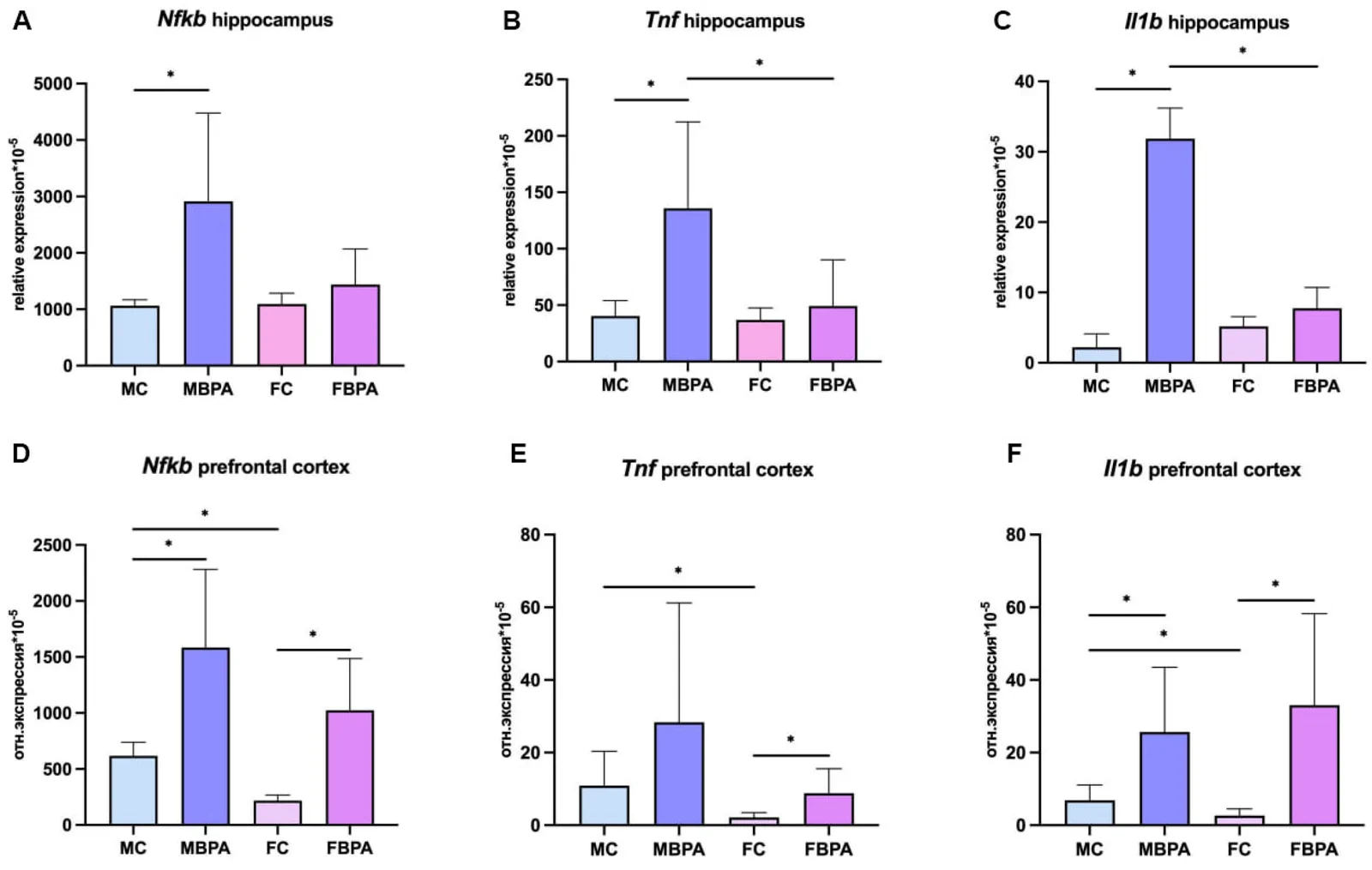
mRNA expression levels of transcriptional master-regulator *Nfkb* (**A**,**D**) and pro-inflammatory cytokines *Tnf* (**B**,**E**) and *Il1b* (**C**,**F**) in the hippocampus (**A**–**C**) and prefrontal cortex (**D**–**F**) of control and experimental newborn male and female rats. MC—males born to control rats; FC—females born to control rats; MBPA—males born to females that received BPA at 30 ng/kg body weight/day from gestational days 6 to 21; FBPA—females born to females that received BPA at 30 ng/kg body weight/day from gestational days 6 to 21. *—*p* < 0.05. Data are presented as Me (LQ 25%; UQ 75%).

To evaluate the effect of BPA on the altered state of microglial cells, we examined the expression levels of the transcription factor *Nfkb*, the pro-inflammatory cytokines *Tnf* and *Il1b*, and the anti-inflammatory cytokine *Tgfb*. NF-κB is a transcription factor associated with the development of inflammatory responses; upon its activation, transcription of genes encoding, among others, the pro-inflammatory cytokines IL-1β and TNF is initiated [53]. NF-κB, TNF, and IL-1β, are also required under physiological conditions for the proliferation and differentiation of neural progenitor cells during embryogenesis and early postnatal neurogenesis [54]. In the hippocampus, only in prenatally BPA-treated males, *Nfkb* expression was markedly upregulated relative to corresponding controls (Figure 9A). There were no differences between the control and experimental groups of females, as well as no sex differences. However, in the prefrontal cortex, the level of *Nfkb* expression showed sex differences and was higher in control males. In offspring of both sexes born to females that received BPA during pregnancy, *Nfkb* expression increased relative to control groups by 1.9-fold in males and 4.3-fold in females, respectively. No sex differences between males and females of the experimental groups were detected (Figure 9D). The mRNA expression level of the pro-inflammatory cytokine *Tnf* also was upregulated in the hippocampus only in the experimental male group compared to controls, with no sex differences detected (Figure 9B). In the prefrontal cortex, there were no sex differences in its expression between control and experimental groups. However, an increase in *Tnf* expression was observed in the prefrontal cortex in both prenatally BPA-treated males and females compared to control groups (Figure 9E). A similar pattern was observed for *Il1b* expression. In both the hippocampus and the prefrontal cortex, its expression was upregulated in prenatally BPA-treated males compared to controls as well as relative to BPA-treated females, whereas it was upregulated only in the prefrontal cortex in experimental females relative to corresponding controls (Figure 9C). Sex differences were identified between control groups, with higher expression levels in males, while no differences were found between experimental groups in the prefrontal cortex (Figure 9F).

**Figure 10:**
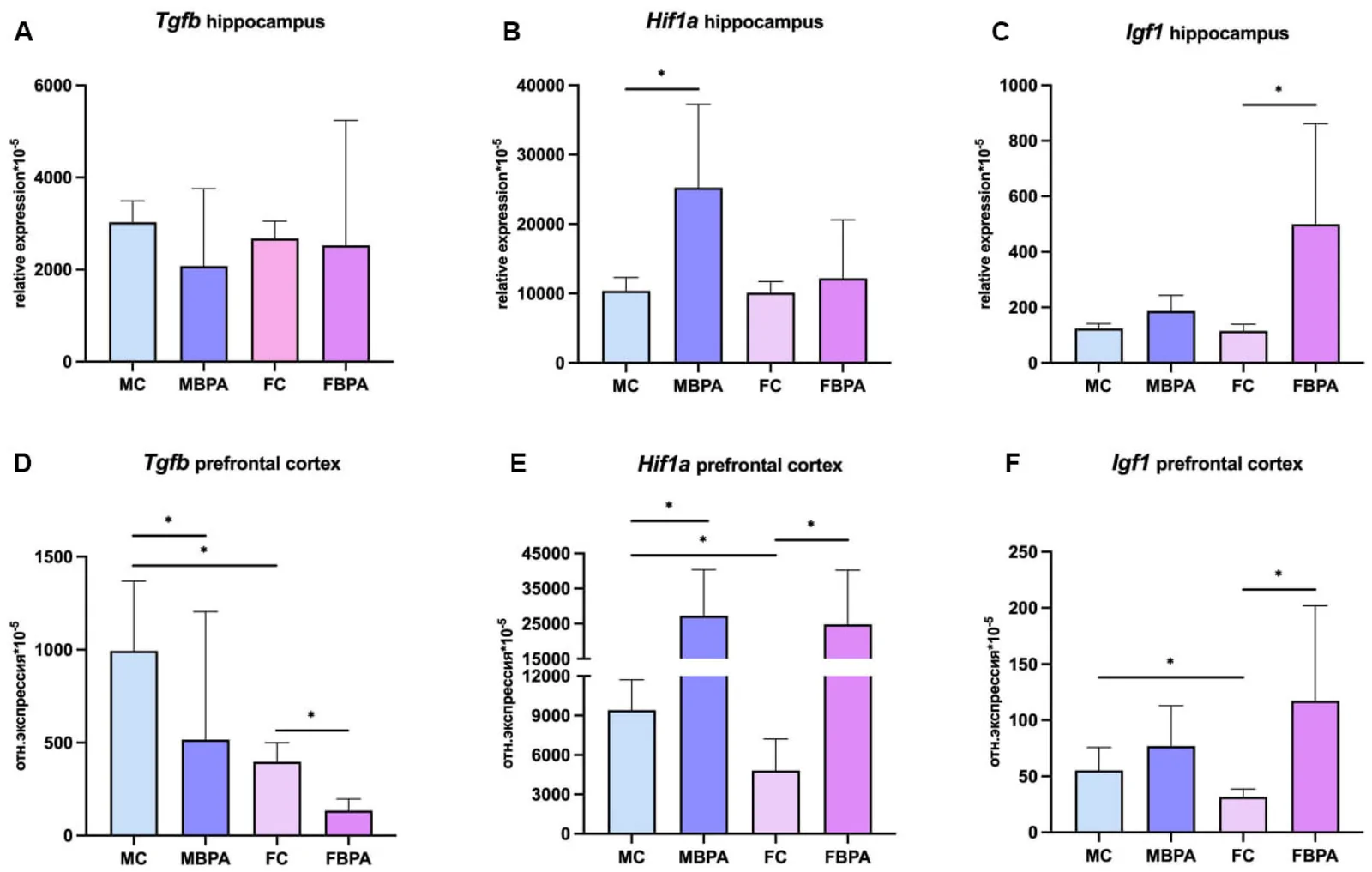
mRNA expression levels of anti-inflammatory cytokine *Tgfb* (**A**,**D**), transcriptional master-regulator *Hif1a* (**B**,**E**), and adaptation marker *Igf1* (**C**,**F**) in the hippocampus (**A**–**C**) and prefrontal cortex (**D**–**F**) of control and experimental newborn male and female rats. MC—males born to control rats; FC—females born to control rats; MBPA—males born to females that received BPA at 30 ng/kg body weight/day from gestational days 6 to 21; FBPA—females born to females that received BPA at 30 ng/kg body weight/day from gestational days 6 to 21. *—*p* < 0.05. Data are presented as Me (LQ 25%; UQ 75%).

TGF-β is secreted by both neurons and glial cells. It regulates microglial activation predominantly toward an anti-inflammatory phenotype by reducing the release of pro-inflammatory cytokines and reactive oxygen species [55]. It is also required for the survival, proliferation, and differentiation of neural progenitor cells [56]. TGF-β exerts mostly anti-inflammatory effects, and its expression and secretion frequently increase simultaneously with pro-inflammatory cytokines via a compensatory mechanism [57]. In our study, no *Tgfb* expression changes were observed due to prenatal BPA exposure in the hippocampus, and there were no sex differences (Figure 10A). In the prefrontal cortex, *Tgfb* expression levels in control groups differed by sex and were higher in males, while no differences were found between BPA-treated males and females; a decrease in *Tgfb* expression was observed in both experimental groups compared to controls. In experimental males, the expression level of this cytokine was 3.8-fold lower, while in females, it was 2.6-fold lower (Figure 10D). The increase in expression of the transcription factor *Nfkb* and the associated pro-inflammatory cytokines *Tnf* and *Il1b* presumably indicates pro-inflammatory activation, primarily of microglia [58]. The concomitant decrease in expression of the anti-inflammatory cytokine *Tgfb* might indicate a predominance of pro-inflammatory reactions and glial activation in the prefrontal cortex, while the hippocampus area is more resilient [55].

The formation of a pro-inflammatory background mediates disruptions in neuron-glial interactions. Consequently, when pro-inflammatory reactions are activated, neuroprotective mechanisms should also be triggered. To assess adaptation to prenatal BPA exposure, we examined the expression levels of the neuroprotective markers *Hif1a* and *Igf1*. HIF-1α plays an important role in embryogenesis and early postnatal neurogenesis, activating the expression of genes responsible for cell proliferation and survival [59]. However, it is also a master-regulator of pro-inflammatory molecular machinery. We identified sex differences in the mRNA expression level of Hif1α in the prefrontal cortex of control group offspring, that is, it was higher in males; no sex differences were observed in *Hif1a* expression in the hippocampus (Figure 10B). Its expression level was upregulated in experimental males compared to controls in both the hippocampus and prefrontal cortex, while in prenatally BPA-treated females, its level was higher only in the prefrontal cortex. (Figure 10E).

*Igf1* exerts neuroprotective effects by inhibiting neuronal apoptosis [60]. Its expression level was also higher in the control male group than in control females in the prefrontal cortex (Figure 10C,F). No sex differences were found in the experimental offspring groups. A 3-fold increase in *Igf1* expression was found only in prenatally BPA-treated females in both hippocampus and prefrontal cortex, while no differences were observed between control and experimental males (Figure 10C,F).

### 3.5. Prenatal BPA Exposure Led to Sex-Dependent Alterations in Neural Progenitor Gene Expression and Protein Level

**Figure 11:**
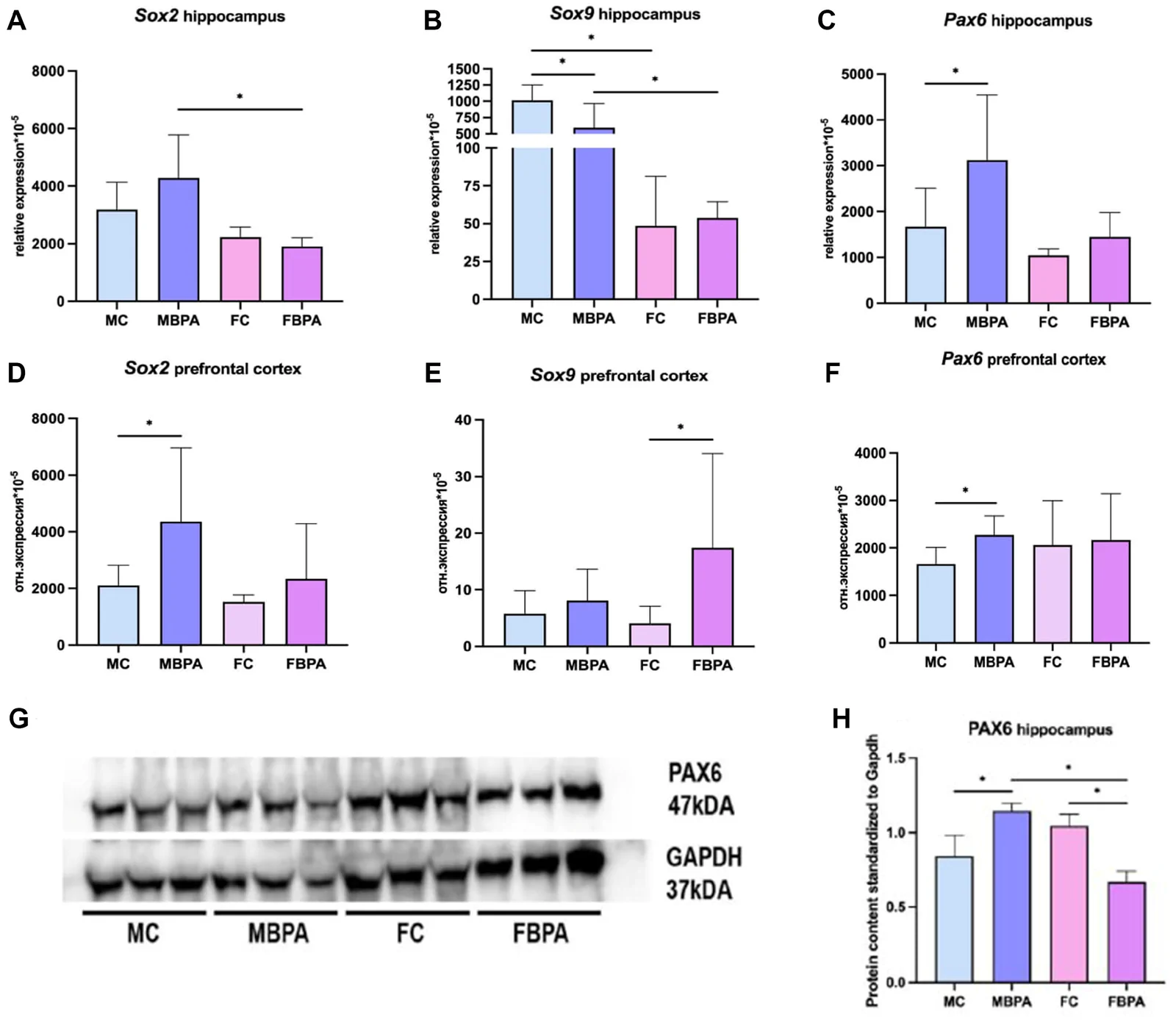
mRNA expression levels of neural progenitor cell markers *Sox2* (**A**,**D**), *Sox9* (**B**,**E**), and *Pax6* (**C**,**F**) in the hippocampus (**A**–**C**) and the prefrontal cortex (**D**–**F**) and Western blotting measurement of PAX6 protein levels in the hippocampus (**G**,**H**) of control and experimental newborn male and female rats. MC—males born to control rats; FC—females born to control rats; MBPA—males born to females that received BPA at 30 ng/kg body weight/day from gestational days 6 to 21; FBPA—females born to females that received BPA at 30 ng/kg body weight/day from gestational days 6 to 21. *—*p* < 0.05. Data are presented as Me (LQ 25%; UQ 75%).

The altered functional state of glial cells, including microglia, has a profound influence on the formation of progenitor niches and the normal differentiation of neural precursors into progenitor cells and mature neurons [61]. To assess the effect of microglial morphofunctional state changes resulting from prenatal BPA exposure on neural stem and progenitor cells, we examined the expression of neural stem cell markers *Sox2* and *Sox9* and the neural progenitor cell marker *Pax6* in the hippocampus and prefrontal cortex, as well as PAX6 protein levels in the hippocampus. In the developing nervous system, *Sox2* is expressed in undifferentiated stem precursor cells, as well as neuronal precursors and astrocytes, and is important for maintaining the progenitor cell pool and differentiation into neurons and glia [62,63]. Sex differences in *Sox2* expression in the hippocampus were observed between experimental groups only. In the prefrontal cortex, no sex differences in *Sox2* expression levels were detected in either control or experimental groups; at the same time, the *Sox2* expression level was 1.8-fold upregulated in prenatally BPA-treated males compared to controls, while it did not change in experimental females (Figure 11A,D). According to the literature, *Sox9* plays an important role in the development and maintenance of the neural stem cell pool and also participates in differentiation [64]. *Sox9* expression showed sex differences between both control and experimental groups in the hippocampus. At the same time, it was downregulated relative to controls only in prenatally BPA-treated males and did not change in prenatally BPA-treated females compared to controls. In the prefrontal cortex, no sex differences in *Sox9* expression levels were detected in either control or experimental groups; the same was true for Sox2. *Sox9* expression was upregulated only in the experimental female group compared to controls, while no differences were found between control and experimental males (Figure 11B,E).

During prenatal and early postnatal ontogenesis, *Pax6* plays a key role in maintaining the multipotent state of brain progenitor cells, participating alongside *Sox2* and *Sox9* in the formation of the progenitor cell niche [65]. As with *Sox2* and *Sox9*, no sex differences in *Pax6* expression were found. Its expression increased only in males born to BPA-treated females in both the hippocampus and prefrontal cortex, but not in females. At the same time, PAX6 protein levels increased in prenatally BPA-treated males’ hippocampi relative to corresponding controls, while in experimental females, the opposite was true (Figure 11G,H). There were sex differences in PAX6 protein levels in prenatally BPA-treated offspring—they were significantly higher in males than in females (Figure 11C,F–H). Changes in gene expression and protein levels of neural stem and progenitor cell markers *Sox2*, *Sox9*, and *Pax6* may reflect differences in the mechanisms of progenitor cell pool generation depending on sex and prenatal BPA exposure.

### 3.6. Social Behavior Patterns Were Altered in Juvenile Offspring of Both Sexes

**Figure 12:**
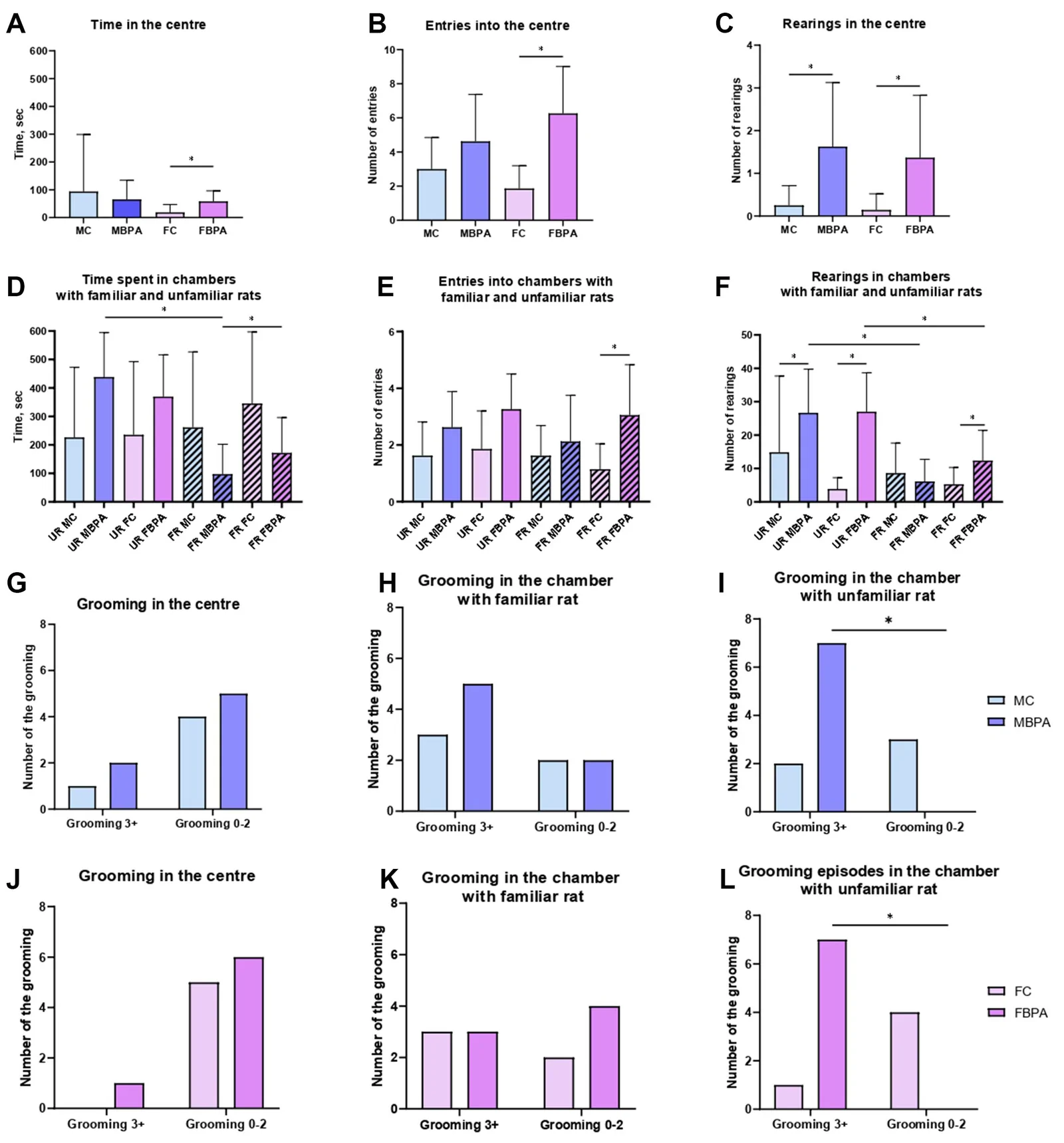
Time (**A**,**D**), the number of entries (**B**,**E**), rearings (**C**,**F**), and grooming episodes (**G**–**L**) in the center (**A**–**C**,**G**,**J**) and chambers with familiar and unfamiliar rats (**D**–**F**,**H**,**I**,**K**,**L**) of control and experimental juvenile male and female rats. MC—males born to control rats; FC—females born to control rats; MBPA—males born to females that received BPA at 30 ng/kg body weight/day from gestational days 6 to 21; FBPA—females born to females that received BPA at 30 ng/kg body weight/day from gestational days 6 to 21. *—*p* < 0.05. Data are presented as Me (LQ 25%; UQ 75%).

Social behavior and novelty preference were assessed in PD21 offspring following BPA treatment using the three-chamber test. Compared with the control groups, both male and female offspring exhibited a significantly higher number of rearings in the center (empty) chamber (Figure 12C) and the chamber with the unfamiliar rat (Figure 12F). Prenatally and during lactation, BPA-treated female offspring were characterized by a higher number of entries in the center (Figure 12A) and more time spent in this section (Figure 12B). They also performed more entries to the chamber with familiar rats relative to control female offspring (Figure 12E) and spent more time in this chamber in comparison with BPA-treated males (Figure 12D). At the same time, only BPA-treated males spent significantly more time with unfamiliar rats relative to familiar ones (Figure 12D). Analysis of grooming behavior revealed that BPA-treated offspring groomed significantly more frequently in the chamber containing the unfamiliar rat than control animals (Figure 12G–L). No other significant differences in behavioral parameters or sex differences were observed.

Observed data are summarized in the Table 1.

## 4. Discussion

During neonatal CNS maturation, the hippocampus and prefrontal cortex undergo vital structural remodeling, marked by the neurogenic-to-gliogenic switch, interneuron integration, and the establishment of adult neural stem cell niches [66]. Concurrently, early synchronous communication between these two regions forms the foundational long-range circuit necessary for future social and cognitive behavior [67]. In ASD, genetic anomalies or pre- and neonatal environmental stressors such as MIA due to BPA exposure might disrupt these synchronized timelines [68]. This could result in an altered glia-to-neuron ratio, deficient synaptic pruning, or their excessive and imperfect formation, as well as an excitation/inhibition imbalance in the hippocampus and the prefrontal cortex [66,69,70]. Subsequently, these molecular and cellular disturbances are potentially able to cause functional desynchronization of the hippocampal–prefrontal network, driving the behavioral, social, and sensory manifestations of ASD [68].

BPA, one of the most widely produced endocrine-disrupting chemicals, is increasingly recognized as an environmental factor capable of altering maternal immune homeostasis during pregnancy. Unlike classical MIA models induced by infectious stimuli such as lipopolysaccharide (LPS) or polyinosinic–polycytidylic acid [poly(I:C)] [71], BPA does not directly activate pathogen recognition pathways. Instead, BPA promotes a state of chronic low-grade inflammation through endocrine disruption, oxidative stress, mitochondrial dysfunction, and activation of inflammatory signaling cascades, particularly the nuclear factor-κB (NF-κB) pathway [72,73,74] as well as through epigenetics via DNA methylation [75]. Experimental and population studies have demonstrated that gestational BPA exposure increases the production of pro-inflammatory cytokines, including interleukin (IL)-1β, IL-6, and tumor necrosis factor-α (TNF-α), in maternal and placental tissues and umbilical blood while impairing immune tolerance at the maternal–fetal interface, as explicitly discussed in the review by Costa H.E. et al. (2025) [72]. This inflammatory intrauterine environment can influence fetal brain development by promoting microglial activation, enhancing oxidative stress, and altering neuronal proliferation, differentiation, and synaptic maturation, thereby increasing susceptibility to long-term neurodevelopmental and behavioral abnormalities in offspring [71,74,76]. Although BPA is not considered a canonical inducer of maternal immune activation, growing evidence suggests that its ability to induce maternal immune dysregulation and fetal pro-inflammatory background formation in the CNS represents an important mechanistic link between prenatal environmental exposure and adverse neurodevelopmental outcomes. Subsequently, it is probable that maternal pro-inflammatory cytokines mediate most observable effects [71], rather than BPA alone. As a result of disruptions to physiological processes during brain maturation, delayed consequences—such as the manifestation of various neuropsychiatric disorders including ASD and ADHD—are likely to emerge in later ontogenesis. One aspect that BPA may adversely affect, particularly at extremely low concentrations, is the maturation and functioning of microglial cells, particularly in relation to their pleiotropic functions and the maintenance of CNS maturation [77,78].

Microglia are immune system cells; unlike astroglia and neurons, they are highly capable of altering their functional state in response to the slightest changes in the microenvironment. Consequently, the pro-inflammatory background naturally affects every cell and their interactions, but it impacts microglia first and foremost. Embryonic microglia can influence the number of neural stem cells in progenitor niches, guide neuronal migration, support the structuring and differentiation of cortical neurons, and promote the formation of synaptic contacts [78]. The colonization of different brain regions by microglia depends on sex and age [79]. At the time of birth (PD0), the hippocampus of female rats typically contains more microglial cells than that of males, whereas by PD4, the number of microglial cells in this region increases in males [80]. In our study, no differences in the total number or in the numbers of amoeboid and ramified Iba1+ microglial cells in the hippocampus were found between control males and females, which may be related to a more intensive colonization of the hippocampus by microglia in males. At the same time, in both experimental groups, the total number of microglial cells was significantly higher, which might be associated with their excessive proliferation and migration. Once microglia are in the parenchyma, they proliferate and expand their population with a peak at two weeks after birth in rodents and decrease in proliferation levels until adulthood [81]. Prenatal BPA consumption has been shown to increase the number of Iba1+ and CD68+ microglial cells in the hypothalamus and other brain regions in E15.5 rats [34,50]; however, in those experiments, pregnant rats received much higher doses of BPA—20 and 200 µg/kg [50] and 50 and 50,000 µg/kg BPA [34]. Furthermore, only male embryos were examined in those studies. We also found that only males that underwent prenatal BPA exposure had a greater number of ramified microglial cells relative to controls, whereas no such differences were found in females. This might indicate a more rapid transition of microglial cells from the immature amoeboid to the mature ramified form, which primarily performs a surveillance function, as well as synaptic pruning and phagocytosis of apoptotic progenitor cells to maintain the stem and progenitor cell population necessary for progenitor niche formation [82]. Consequently, neuronal migration in the hippocampus of males is potentially compromised, and pruning and phagocytosis might be excessive due to the earlier shift in the morphofunctional status of microglial cells from amoeboid to ramified.

The microenvironment in progenitor niches, such as the periventricular zone, is critically important for maintaining normal neurogenesis throughout life [83]. It has been shown that newborn male Sprague–Dawley rats are characterized by a greater number of Iba1+ cells in the periventricular zone than females [79], which we also found in our study. However, in contrast to the hippocampus, in the periventricular zone, the total number of Iba1+ microglial cells in offspring of both sexes born to BPA-treated females showed a tendency toward reduction. At the same time, the number of amoeboid Iba1+ cells in experimental groups showed sex differences and was significantly lower in males compared to controls, while no differences were observed in females. This is possibly related to the migration of amoeboid microglia from the periventricular zone to other brain regions, which potentially could cause a disruption of neuronal migration and progenitor cell pool formation. Furthermore, despite the absence of statistically significant differences, experimental males showed a tendency toward increased *Aif1* expression relative to the corresponding controls, while experimental females showed a tendency toward decreased expression. Activation of *Aif1* expression and an increase in Iba1 protein levels correlate with microglial polarization toward a pro-inflammatory phenotype, as previously shown by Zhao J. et al. (2019) [52]. At the same time, according to Gheorghe R.O. et al. (2020), blocking Iba1 with siRNA in BV2 mouse microglial cells led to a reduction in their migratory capacity but activated phagocytosis [84]. Our data allow us to suggest that even extremely low doses of BPA, approximately corresponding to the average global daily intake of an adult human [3], affect the number and morphofunctional characteristics of microglial cells in a sex-dependent manner: changes observed in males following prenatal BPA exposure are more pronounced, potentially indicating greater vulnerability of their developing CNS to the effects of BPA.

The transcriptional landscape and its disturbances in both the hippocampus and the prefrontal cortex are pivotal factors affecting the possibility of ASD development [85]. Expression levels of *Esr1*, encoding ERα, in the prefrontal cortex of males and females that underwent prenatal BPA exposure were reduced compared to the corresponding control groups and were higher in males than in females in both control and experimental groups. At the same time, expression of *Esr2*, encoding ERβ, displayed opposite dynamics between control and prenatally BPA-treated offspring of both sexes. At the same time, prenatally BPA-treated males showed *Esr1* downregulation in the hippocampus. However, in females, *Esr2*. Erα, and ERβ are critically required for estrogen-dependent neuronal survival and normal brain maturation, including through the inhibition of Akt/NF-κB signaling mediated by activation of the membrane G protein-coupled estrogen receptor GPER1 [86]. Estrogen, through its receptors and the associated G protein, regulates dopamine D1 and D2 and serotonin 5HT2A and 5HT2C receptors [87], inhibiting the reuptake of dopamine and serotonin as well as serotonin breakdown by monoamine oxidase [88] and activating serotonin synthesis from tryptophan [89]. Estrogen also mediates the expression and secretion of anti-inflammatory mediators and protects neurons—particularly cortical and hippocampal neurons—from glutamate excitotoxicity [90]. Previously, upregulation of both *Esr1* and *Esr2* was described in prenatally BPA-treated male and female offspring in the amygdala; however, the doses of BPA were higher, at 2.5 and 25 µg/mother body weight/day, DG6-21 [91]. In our study, experimental male offspring exhibited a more pronounced imbalance in *Esr1* and *Esr2* expression in the hippocampus and the prefrontal cortex relative to females. A disruption in estrogen receptor expression and the consequent suppression of estrogenic signaling in the brain correlate with the development of neuropsychiatric diseases at various stages of ontogenesis, e.g., ASD and ADHD in early childhood and pre-puberty; eating disorders, generalized anxiety disorder, bipolar disorder, and major depressive disorder in puberty and young adulthood [92]; and neurodegenerative diseases including Alzheimer’s disease in older and elderly age [93]. *Esr1* and *Esr2* imbalances, which are more pronounced in prenatally BPA-treated males, might lead to delayed negative consequences associated with reduced stability of neuronal networks between the hippocampus and the prefrontal cortex (which are key brain regions regulating behavioral responses) [94].

According to the literature, BPA, even at extremely low concentrations (down to 50 ng/L), activates the production of pro-inflammatory cytokines in many cell types, including in the brain [95,96]. In our study, we demonstrated that the expression level of the transcription factor *Nfkb* and the pro-inflammatory cytokines *Il1b* and *Tnf*—whose protein products are synthesized and released by microglia and astrocytes [97]—was elevated in the prefrontal cortex of experimental offspring of both sexes relative to corresponding control groups. At the same time, in control males, the expression of all these genes was higher compared to females, which might reflect a more pronounced pro-inflammatory background, which could potentially promote microglial cell activity. Further, prenatally BPA-treated females exhibited only *Il1b* upregulation in the hippocampus. Simultaneously, in both experimental groups, the expression of the anti-inflammatory *Tgfb* was reduced in the prefrontal cortex but remained unchanged in the hippocampus; its level was also higher in control males relative to females. Moreover, in prenatally BPA-treated males, its expression level showed a greater decrease of 3.8-fold compared to control males, while in experimental females, it showed a 2.6-fold decrease compared to controls. In neonatal brains, these molecules participate in normal synaptic development, neuronal differentiation, gliogenesis, and developmental remodeling. However, the balance between pro- and anti-inflammatory cytokines plays an important role in the development of inflammatory responses; if, upon an increase in pro-inflammatory cytokines, the production of anti-inflammatory cytokines undergoes a compensatory increase, the balance is maintained [98]. In cases where the expression of several pro-inflammatory cytokines predominates over anti-inflammatory ones, as demonstrated in our experimental offspring of both sexes, the inflammatory process might be exacerbated, including through the altered functional state of microglial cells, which are critical for the developing brain.

The mRNA expression level of *Hif1a* in the prefrontal cortex was elevated in both males and females upon prenatal BPA exposure, while *Igf1* expression was significantly higher only in experimental females relative to controls, with experimental male offspring showing only a tendency toward its elevation compared to control males. Moreover, *Igf1* expression was upregulated in the hippocampus only in prenatally BPA-treated females, while males exhibited only *Hif1a* upregulation. No data in the literature are available regarding the effect of prenatal BPA consumption, especially at low doses, on *Hif1a* and *Igf1* expression levels. It is known that postnatal BPA consumption at high doses of 5 and 25 mg/kg for 40 days leads to an increase in HIF-1α content in the hippocampus [99]. HIF-1α plays an important role during early postnatal development in regulating the adaptive response to hypoxia, activating the expression of genes responsible for cell proliferation and survival, energy metabolism, and glucose and iron metabolism [59]. However, it is also a master-regulator of pro-inflammatory molecular machinery. Regarding upregulation of its pro-inflammatory targets such as *Il1b*, *Tnf*, and *Nfkb* itself, we presume it is involved in the formation of a pro-inflammatory background in experimental male offspring, rather than in protection, as shown before in neonatal brain injury model [100]. IGF-1 is a critical regulator of normal brain development; in addition, it exerts neuroprotective effects by inhibiting neuronal apoptosis [60]. Huang B. et al. (2025) showed that when neural progenitor cells derived from human embryonic stem cells were cultured in the presence of BPA, *Igf1* expression was reduced, as was the expression of the progenitor marker *Sox5*, which positively regulates *Igf1* expression [101]; however, the BPA dose used in that study was relatively high. The increase in *Igf1* expression observed in prenatally BPA-treated female offspring apparently reflects adaptive processes that are activated to compensate for the negative effects of BPA. It can therefore be suggested that females are more resilient to prenatal BPA exposure.

Despite the general tendencies toward increased expression levels of the stem cell markers *Sox2* and *Sox9* and the neural progenitor cell marker *Pax6* in the prefrontal cortex of neonatal males and females that underwent prenatal BPA exposure, *Sox2* and *Pax6* expression was significantly higher only in experimental males, while *Sox9* was elevated only in experimental females. At the same time, prenatally BPA-treated males exhibited downregulation of *Sox9* alongside with upregulation of *Pax6* and an increase in PAX6 protein levels in the hippocampus, whereas there was a decrease in PAX6 protein levels in prenatally BPA-treated females. The increase in *Pax6* expression beyond the progenitor niches in males may indicate excessive migration of immature progenitor cells into the prefrontal cortex [65], which might negatively affect the further maturation and differentiation of neurons in this brain region. PAX6 and SOX2 have opposing effects: PAX6 initiates further differentiation of progenitor cells into neuronal progenitor cells, while SOX2 maintains them in a pluripotent state [102]. Consequently, the balance between PAX6 and SOX2 is critical to ensure accurate progenitor cell specification. It should be noted that elevated *Sox2* and *Pax6* expression may be associated with a proliferative phenotype in neural progenitors and the development of ASD in humans [103]. The simultaneous upregulation of *Sox2* and *Pax6* in males that underwent prenatal BPA exposure likely reflects the preservation of this balance; however, further studies are required, including assessment of both PAX6+ and SOX2+ cell numbers. As an increase in PAX6 protein levels potentially indicated an imbalance in neural progenitors’ maturation in prenatally BPA-treated offspring, this could result in progenitor niche formation disruption and the manifestation of neuropsychiatric disorders in late ontogenesis [83]. SOX9 is also a marker of progenitor cells and regulates their differentiation predominantly toward astrocytes [104,105]. It has been shown that lipopolysaccharide (LPS)-induced activation of MIA in late gestation reduced *Sox9* expression in the prefrontal cortex of newborn females compared to males [31]. BPA entering the body during pregnancy may also trigger MIA, which in turn may lead to the development of various neuropsychiatric diseases during different periods [71]. Although BPA, like LPS, induces microglial cell activation and the formation of a pro-inflammatory background in the brain, the low dose of BPA used in our study apparently initiated differentiation of progenitor cells in experimental females toward astrocytes—the primary cells supporting neuronal survival and providing neuroprotection. The downregulation of *Sox9*, upregulation of *Pax6*, and increase in PAX6 protein levels in the hippocampus of male offspring could potentially ensure more stable neuronal function and a lower risk of the clinical manifestation of neuropsychiatric disorders in females.

MIA is a well-established experimental paradigm for investigating how prenatal inflammatory exposure may contribute to neurodevelopmental alterations associated with potential development of neuropsychiatric disorders such as ASD and ADHD [71,106]. Behavioral abnormalities induced by MIA are highly dependent on the developmental stage of the offspring. As previously described, while adult animals frequently exhibit reduced sociability and impaired social novelty preference, juvenile animals often display increased exploratory activity, abnormal behavioral flexibility, and dysregulated responses to novel social stimuli rather than social avoidance [106]. In our study, juvenile (PD21) offspring of both sexes treated with BPA prenatally and during lactation demonstrated an increased number of rearings in the center (empty chamber) and the chamber with unfamiliar rats as well as grooming episodes in the chamber with unfamiliar rats. These findings are consistent with altered exploratory activity and behavioral reactivity, although the underlying mechanisms cannot be determined from the present behavioral paradigm. The increased rearing behavior might reflect an altered response to environmental novelty. Rearing is generally considered an index of environmental investigation and behavioral activation [107]. Increased vertical exploration has been associated with elevated arousal and altered processing of novel environments in juvenile rodents rather than improved cognitive performance [108]. It might also be connected with altered hippocampal function, including increased noradrenergic transmission in this structure [108], which is vital for spatial orientation in rodents [109]. However, whether such mechanisms contribute to the behavioral changes observed here is unknown for now. The increase in grooming episodes, specifically in the chamber with the unfamiliar rat, might presumably indicate enhanced emotional reactivity during social exposure. Altered grooming has also been used in some experimental paradigms as an indicator of repetitive or stereotyped behavior [110]. However, increased grooming alone is not sufficient to establish repetitive-behavior phenotypes relevant to ASD. Thus, increased grooming in the presence of an unfamiliar rat probably reflects altered emotional regulation rather than enhanced affiliative behavior.

Notably, only female offspring treated with BPA prenatally and during lactation were characterized by an increased number of entries in the chamber with familiar rats as well as an increased number of entries and time spent in the center. Since exploratory transitions increased in both social and non-social compartments, the elevated entries into both the social and empty chambers likely reflect enhanced exploratory drive rather than increased social motivation. This interpretation is consistent with recommendations for the three-chamber test analysis, where chamber entries should be interpreted together with locomotor activity and time spent investigating the stimulus animal [111]. The observed behavioral pattern may potentially overlap with behavioral features reported in previously experimental models relevant to ADHD, particularly increased exploratory or locomotor activity. Hyperactivity, increased locomotion, impulsive exploration, and elevated novelty seeking are hallmark characteristics of several genetic and environmental ADHD models, including juvenile spontaneously hypertensive rats (SHRs) and dopamine transporter-deficient animals [112,113]. Epidemiological studies further demonstrate considerable overlap between ASD and ADHD, with both disorders involving dysfunction of frontostriatal and corticostriatal circuits regulating attention, behavioral inhibition, and social behavior [114]. The observed upregulation of pro-inflammatory genes in the prefrontal cortex provides a potential molecular correlate of the behavioral alterations observed in juvenile offspring. However, a causal relationship between these molecular and behavioral changes cannot be established from the present study.

The juvenile age of the offspring included in the behavioral study should also be considered when interpreting these findings. Neural circuits involved in executive control, social cognition, and inhibitory regulation continue to mature throughout adolescence. Thus, behavioral alterations at PD21 may represent an early developmental phenotype characterized by increased exploration and behavioral dysregulation, which later progresses toward the reduced sociability and repetitive behaviors more commonly reported in adult MIA offspring [106].

To facilitate interpretation of these results, it is important to distinguish between the study’s primary findings and exploratory observations. Primary findings are the predefined results that directly address the main research objectives and hypotheses, providing the principal evidence on which the study’s conclusions are based. In our study, prenatal exposure to a globally relevant dose of BPA was associated with sex-dependent changes in microglial state, which modify the expression of genes associated with pro-inflammatory background and neurodevelopment. Additionally, differences in behavioral responses during the juvenile period were observed. The exploratory observations comprise additional findings that provide mechanistic insight. Such observations include region-specific and sex-specific differences in the expression of individual molecular markers, including estrogen receptors, neural progenitor markers, and neuroprotective genes, as well as the specific behavioral features discussed above. These findings should be regarded as hypothesis-generating and interpreted with appropriate caution until confirmed in independent studies.

## 5. Limitations

Several limitations of the present study should be acknowledged when interpreting the findings. First, while prenatal exposure was established through maternal administration, the precise concentration of BPA in the offspring serum was not precisely detected due to its extremely low level. Consequently, we could not determine the exact internal dose reaching the fetal or neonatal systemic circulation, nor could we establish a direct correlation between circulating BPA and the observed neurobiological changes. However, we identified changes in microglia numbers and characteristics, as well as transcriptional alterations in the hippocampus and the prefrontal cortex in both sexes of the prenatally BPA-treated offspring. These results may be attributed to MIA and the effects of mothers’ pro-inflammatory cytokines.

Second, this study focused predominantly on newborn offspring, providing a cross-sectional snapshot of the immediate postnatal period. Because the neonatal brain is in a highly dynamic state of development, it remains unclear whether the observed alterations in microglial morphology, estrogen receptor expression, and neural progenitor differentiation represent permanent structural deficits or transient developmental delays. Only a behavioral study involving juvenile offspring has been performed thus far. Longitudinal studies extending into adolescence and adulthood are required to determine the long-term functional and behavioral trajectories of these neonatal changes. Furthermore, according to the 3R principle, a limited number of litter units were investigated.

Third, the behavioral assessment was limited to a three-chamber social interaction test performed during the juvenile period. Although this paradigm provides information on social approach and preference between familiar and unfamiliar rats, the present experimental design did not include a non-social empty-cage condition or a separate assessment of sociability. Therefore, the observed differences were not interpreted as definitive evidence of impaired sociability or broader cognitive dysfunction. Moreover, behavioral parameters such as increased chamber entries, center exploration, and grooming might be influenced by general locomotor activity, exploratory drive, novelty seeking, anxiety-like responses, or altered reactivity to social stimuli. In addition, because the behavioral assessment was performed at a single developmental time point, it remains unclear whether these alterations persist into adolescence or adulthood or are associated with resilient behavioral alterations. Future studies should incorporate complementary cognitive paradigms, such as spatial learning and memory or recognition-memory tasks, together with a complete three-chamber protocol including both sociability and social novelty phases, to distinguish alterations in social behavior from broader cognitive and exploratory phenotypes.

Finally, while we documented robust transcriptional alterations in pro-inflammatory (*Nfkb*, *Tnf*, *Il1b*, *Hif1a*) and anti-inflammatory (*Tgfb*) pathways, our analysis was restricted to gene expression at the mRNA level. Given the complex nature of post-transcriptional regulation and translation efficiency—as highlighted by the divergent Pax6 mRNA and protein profiles observed in this study—further proteomic confirmation via Western blotting or ELISA is necessary to fully validate the functional inflammatory state of the neonatal brain. Overall, taking into account all the aforementioned restrictions, the present manuscript should largely considered exploratory research.

## 6. Conclusions

Taken together, our findings suggest that extremely low doses of BPA presumably lead to MIA that persists throughout lactation. The neonatal male brain responds to prenatal BPA exposure with an altered microglial profile, which presumably shifts to a pro-inflammatory state, and a pronounced imbalance in the expression of *Esr1* and *Esr2*. The increased PAX6 protein levels in the hippocampus of males might indicate an excessive progenitor differentiation, which in future could lead to disturbances in neural network formation. The female brain, by contrast, displayed pro-inflammatory cytokine upregulation mostly in the prefrontal cortex and restricted inflammatory signaling in the hippocampus; in prenatally BPA-treated females, estrogen receptor expression was significantly less pronounced relative to males. This was accompanied by upregulation of neuroprotective *Igf1* and astrocyte progenitor *Sox9*. At the same time, a decrease in PAX6 protein level in experimental females might be associated with the dysfunction of neurogenic niches. These baseline neonatal differences underscore the necessity of sex-specific approaches when studying early-life neurodevelopment, inflammatory response, and subsequent behavioral vulnerabilities in different periods of adulthood. They were followed by behavioral changes during early postnatal development, producing a phenotype characterized by hyper-exploration, increased environmental investigation, and heightened emotional reactivity during social exposure, which were more pronounced in female offspring. Such changes did not only occur due to prenatal exposure, but were also due to exposure in lactation, which corresponds to human BPA exposure as well. These alterations are compatible with early manifestations of neurodevelopmental dysfunction and may represent a developmental precursor of the more pronounced behavioral abnormalities observed later in life. The present findings provide exploratory evidence for a potential association between prenatal and lactation BPA exposure, pro-inflammatory signaling, and altered social behavior and also underscore the need for additional studies on the effects of low-dose prenatal BPA exposure.

## Figures and Tables

**Table 1:** Molecular, morphological, and behavioral changes in prenatally BPA-treated offspring of both sexes compared to control groups.

MBPA	FBPA
**Newborn offspring**	
**Iba1+ microglia in the hippocampus**	
Increased total number of cells Increased number of ramified cells	Increased total number of cells
**Iba1+ microglia in the periventricular zone**	
Decreased number of ameboid cells	
**Gene expression in the hippocampus**	
Upregulation of microglia marker *Aif1* Upregulation of estrogen receptor *Esr1* Upregulation of pro-inflammatory *Nfkb*, *Tnf*, *Il1b*, and *Hif1a* Upregulation of neural progenitor cells marker *Pax6* Downregulation of neural stem cells marker *Sox9*	Upregulation of neuroprotector *Igf1* Downregulation of estrogen receptor *Esr2*
**Gene expression in the prefrontal cortex**	
Upregulation of pro-inflammatory markers *Nfkb, Tnf*, *Il1b*, and *Hif1a* Upregulation of neural stem cells marker *Sox2* and neural progenitor cells marker *Pax6* Downregulation of estrogen receptor *Esr2* Downregulation of anti-inflammatory cytokine *Tgfb*	Upregulation of estrogen receptor *Esr2* Upregulation of pro-inflammatory *Nfkb*, *Tnf*, *Il1b*, and *Hif1a* Upregulation of neural stem cells marker *Sox9* Upregulation of neuroprotector *Igf1* Downregulation of estrogen receptor *Esr1* Downregulation of anti-inflammatory cytokine *Tgfb*
**PAX6 protein level in the hippocampus**	
Increased	Decreased
Juvenile offspring	
Behavioral study for social preference and novelty	
Increased number of rearings in the center and the chamber with unfamiliar rats Increased grooming episodes in the chamber with unfamiliar rats	Increased number of rearings in the center and the chamber with unfamiliar rats Increased grooming episodes in the chamber with unfamiliar rats Increased entries in the chamber with familiar rats Increased number of entries and time spent in the center

## Data Availability

All data analyzed during this study are included in this article and are available from the corresponding author upon reasonable request.

## Supplementary Materials

The following supporting information can be downloaded at https://www.mdpi.com/article/10.3390/life16091526/s1, Supplement S1: Primers used for qPCR-RT (*Rattus norwegicus*).
