# Supplementary material for: Prenatal and Lactation Exposure to Bisphenol A (BPA) at the Global Mean Daily Intake Level Leads to Sex-Dependent Changes in Microglial State, Gene Expression, and Behavioral Patterns in Newborn and Juvenile Wistar Rat Offspring
Source: Life (Basel). 2026 Sep 14;16(9):1526. doi: 10.3390/life16091526 (PMC13608422; doi:10.3390/life16091526)
Supplement: Supplementary file 1 [file life-16-01526-s001.zip › life-4437590-supplementary.pdf]

Supplement S1.

Primers used for qPCR-RT (Rattus norwegicus).

Gene	Forward sequence	Reverse sequence
Esr1	ATCCACACACGCTCTGCCTT	GGTACAGACTGGCTTCCCGC
Esr2	TTGCAGAAGCCAAGGGGTACAT	CTGGACAGGGATGAGGGGAAG
Sox2	TCCATGGGCTCTGTGGTCAA	CATGTGCAGTCTACTGGGCG
Sox9	AGAGGCCACCGAACAGACT	TGCTCAGCTCACCGATGTC
Nfkb	AGAGCAACCGAAACAGAGAGG	TTTGCAGGCCCCACATAGTT
Il1b	CTGTCTGACCCATGTGAGCT	ACTCCACTTTGGTCTTGACTT
Tgfb	CCGCAACAACGCAATCTATG	AGCCCTGTATTCCGTCTCCTT
Hif1a	TCACAGTCGGACAACCTCAC	TGCTGCAGTAACGTTCCAATTC
Tnfa	CCACCACGCTCTTCTGTCTA	GCTACGGGCTTGTCACTCG
Aif1	AGCAAGGATTTGCAGGGAGG	TGAAGGCCTCCAGTTTGGAC
Gapdh	GCGAGATCCCGCTAACATCA	CCCTTCCACGATGCCAAAGT
